# Supplementary figures and images for: Regulation of gene expression downstream of a novel Fgf/Erk pathway during Xenopus development
Source: PLoS One. 2023 Oct 19;18(10):e0286040. doi: 10.1371/journal.pone.0286040 (PMC10586617; doi:10.1371/journal.pone.0286040)

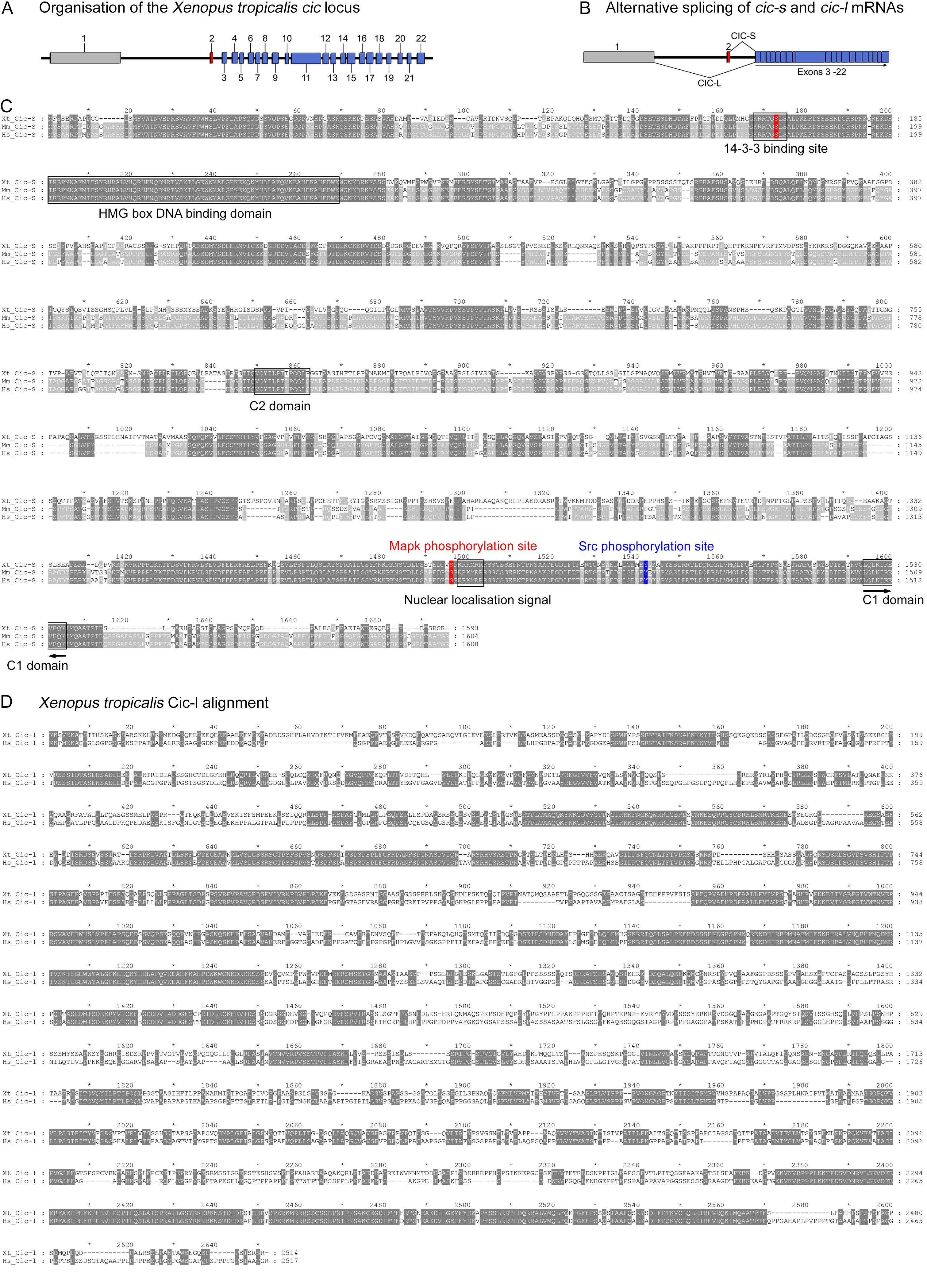

Supplement: S1 Fig — A, diagram of the exon/intron structure of the Xenopus cic locus. B, diagram showing how alternative splicing of exons 1 and 2 give rise two Cic isoforms with different N-termini. C, Conceptual Xenopus Cic-s peptide sequence aligned with Cic-s from human and mouse. The conserved HMG box DNA binding domain, potential monopartite nuclear localisation signal, C1 repression domain, C2 Mapk docking domain, Mapk and Src phosphorylation sites are indicated. D, peptide sequence alignment of Xenopus and human Cic-l. (JPG) [file pone.0286040.s001.jpg]

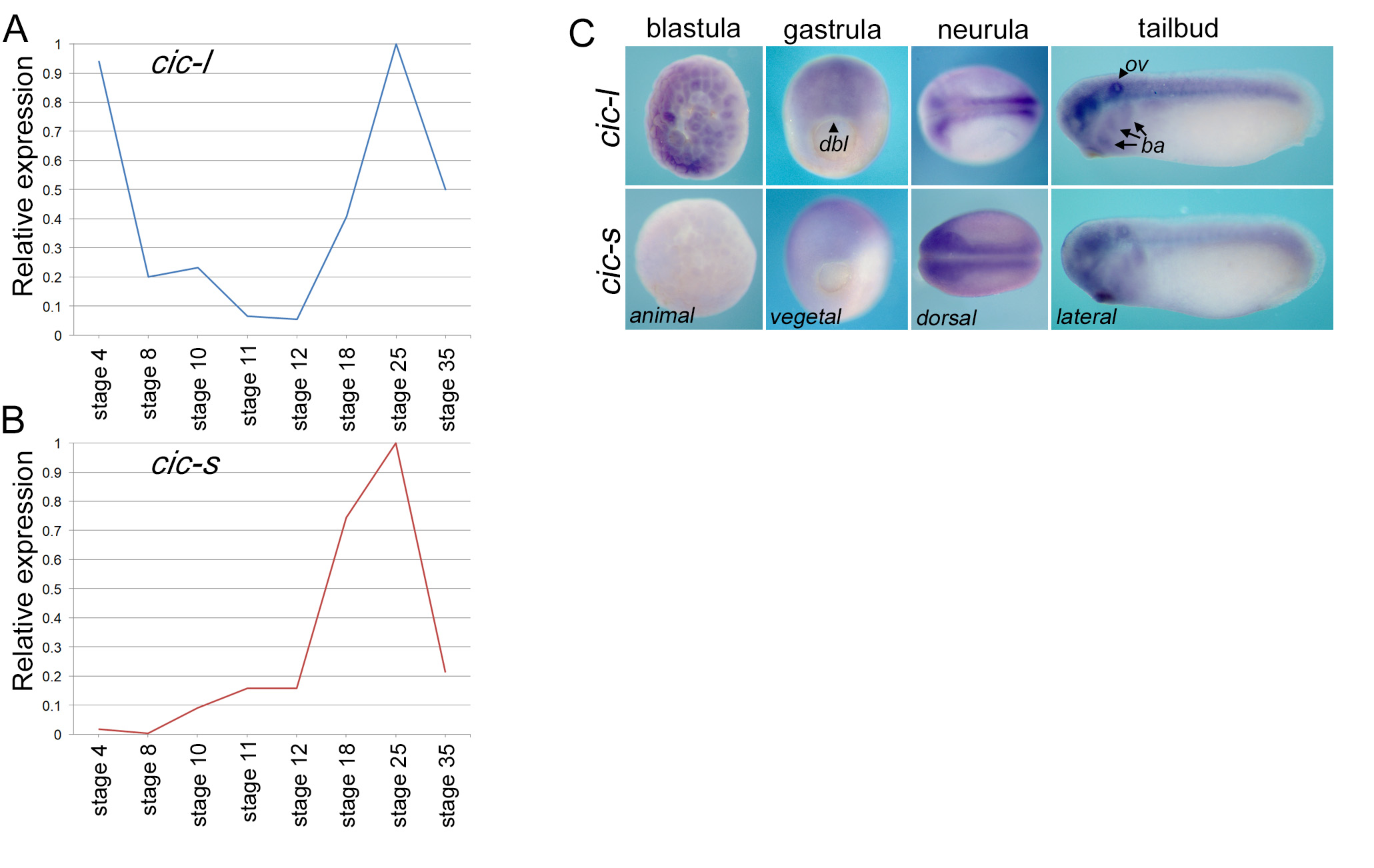

Supplement: S2 Fig — A and B, qPCR analysis of cic-l and cic-s. Expression is normalised to ubiquitously expressed control dicer and shown as relative to maximum expression level. C, in situ hybridisation analysis using isoform specific antisense probes to cic-s and cic-l. Embryo orientations are indicated. dbl = dorsal blastopore lip, ov = otic vesicle and ba = branchial arches. (JPG) [file pone.0286040.s002.jpg]

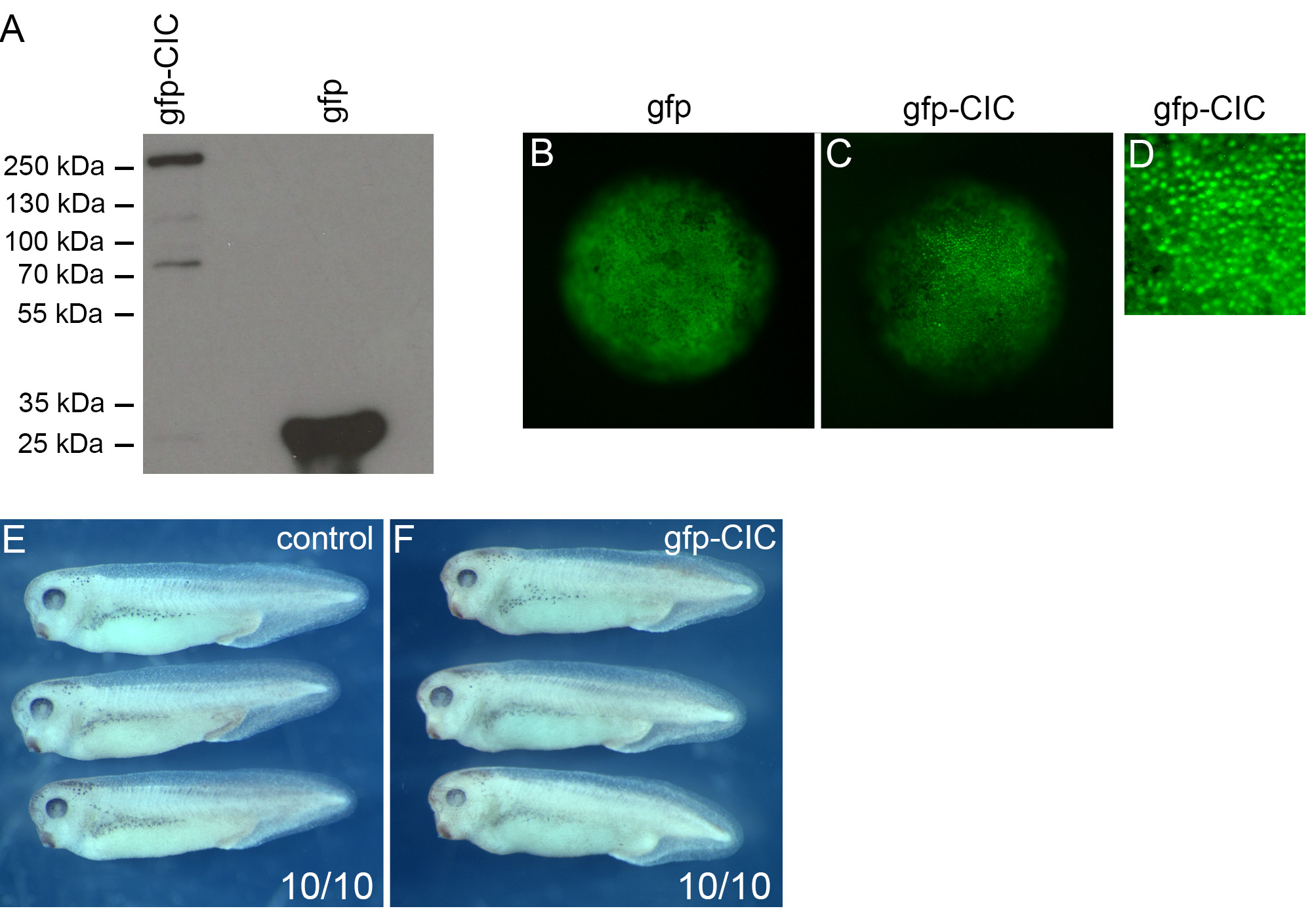

Supplement: S3 Fig — A, is a western blot detecting gfp (~27 kDa) and gfp-tagged human CIC (~250 kDa) proteins translated in gastrula stage embryos from injected mRNAs (image is uncropped). B, shows fluorescence of cytoplasmic gfp protein in the animal hemisphere of a gastrula stage embryo. C and D (higher magnification), show strong nuclear expression of gfp-CIC. E, phenotype at larval stage 37 of uninjected control embryos and F, embryos injected with 6ng synthetic gfp-CIC mRNA. NB Indicated n values are from a representative experiment. (JPG) [file pone.0286040.s003.jpg]

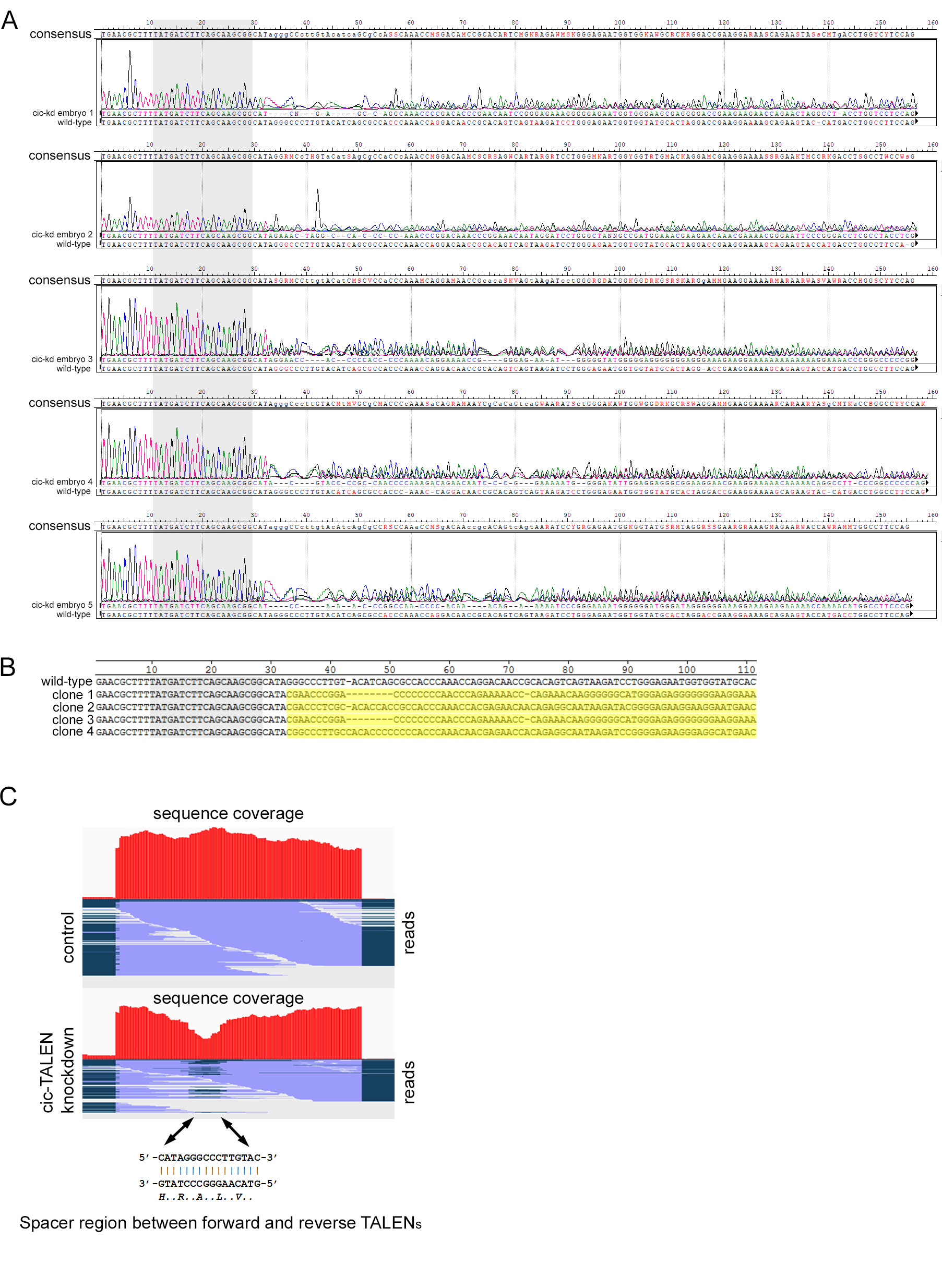

Supplement: S4 Fig — A, sequencing traces of an amplicon from the region of Cic exon 6 targeted by the Cic TALEN pair using genomic DNA from individual knockdown embryos as template. Wild-type sequence is included as a comparison. Grey block indicates sequence bound by the Cic forward TALEN. B, sequence of individual clones obtained from cloning exon 6 amplicon from an individual knockdown embryo. Yellow shading indicates region of sequence mismatches with wild-type exon 6 sequence. C, individual RNA-seq reads from control water injected embryos and Cic-TALEN injected embryos mapped to exon 6 of the cic locus. Combined BAM files from the three experimental replicates were mapped to the Xenopus tropicalis genome using the IGV browser and the output adapted. Dark grey indicates sequence mismatches. The spacer region between the binding sites of the forward and reverse TALENs is indicated. (JPG) [file pone.0286040.s004.jpg]

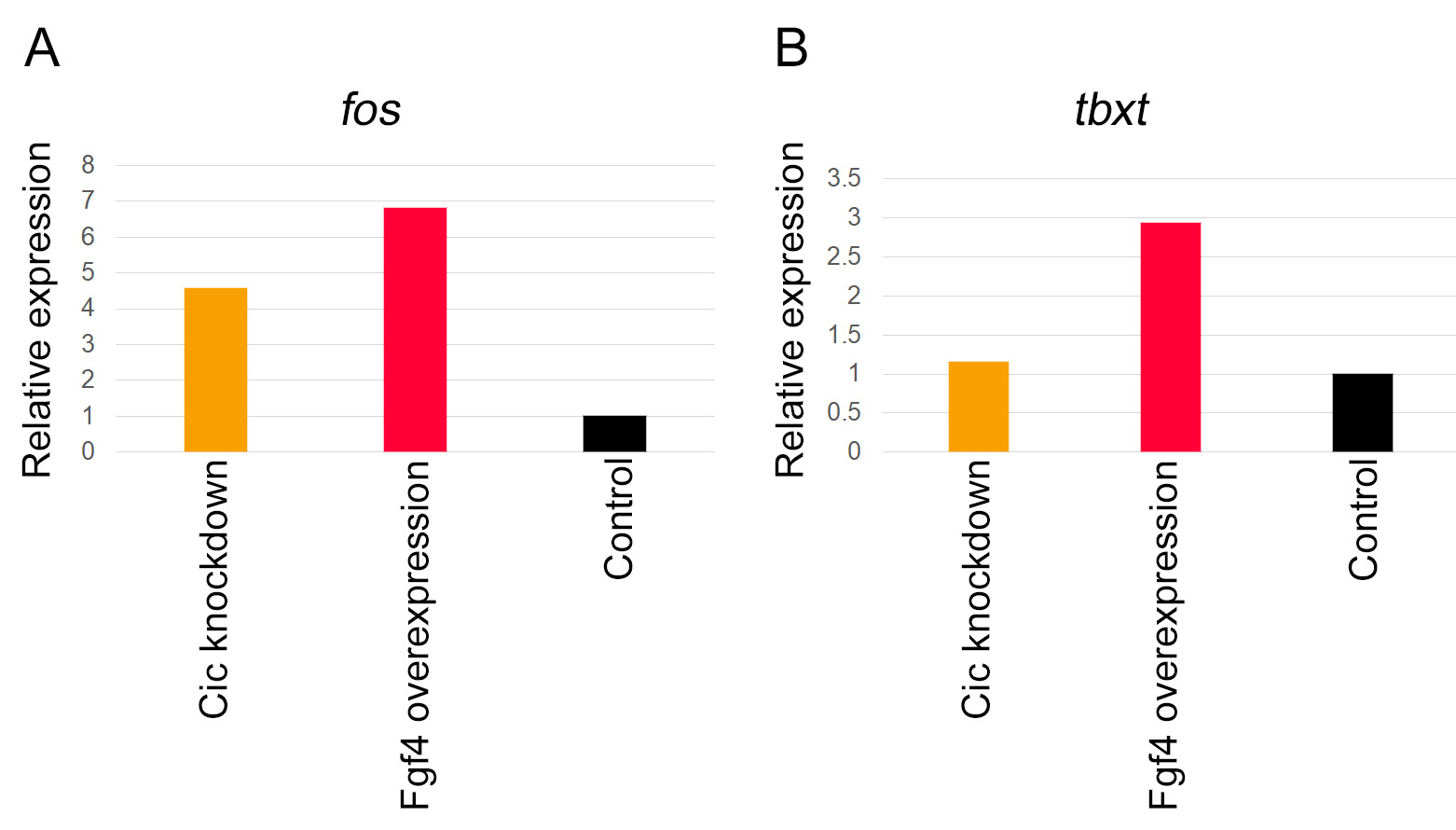

Supplement: S5 Fig — A and B, qPCR analysis of fos and tbxt mRNA expression in Fgf4 overexpressing, Cic knockdown and control water injected embryos at neurula stage 14. Values are normalised to dicer mRNA expression and shown relative to expression in control embryos. (JPG) [file pone.0286040.s005.jpg]

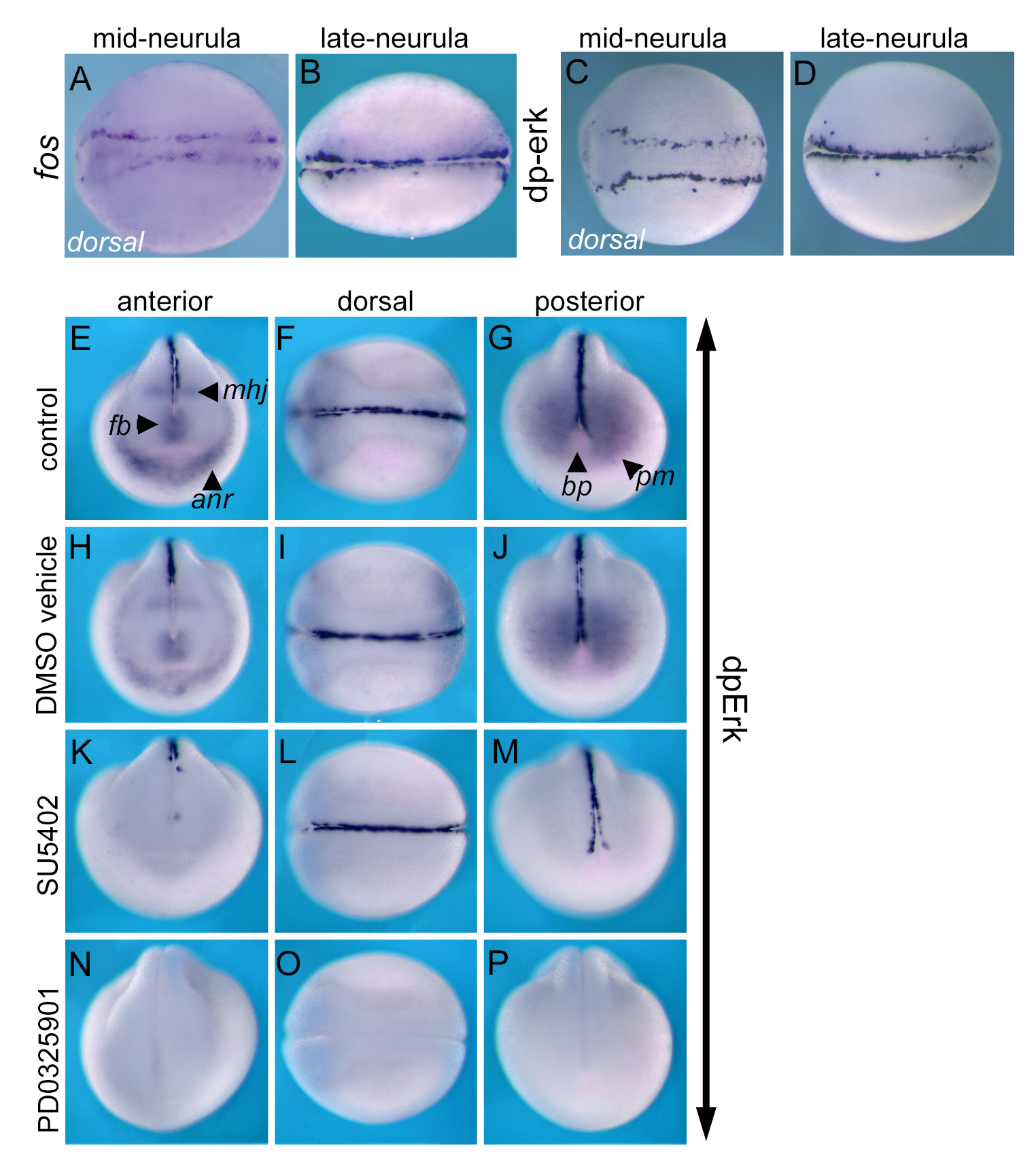

Supplement: S6 Fig — Dorsal views of neurula stage embryos showing localisation of fos mRNA by in situ hybridisation, A and B, and dpErk by immunohistochemistry, C and D. E-P, immunolocalisation of dpErk in untreated control embryos and embryos treated with 0.1% DMSO vehicle, 200 μM Fgfr inhibitor SU5402 or 25 μM Mek inhibitor PD0325901. anr = anterior neural ridge, bp = blastopore, fb = forebrain, mhj = midbrain/hindbrain border, pm = posterior mesoderm. NB Indicated n values are from a representative experiment. (JPG) [file pone.0286040.s006.jpg]

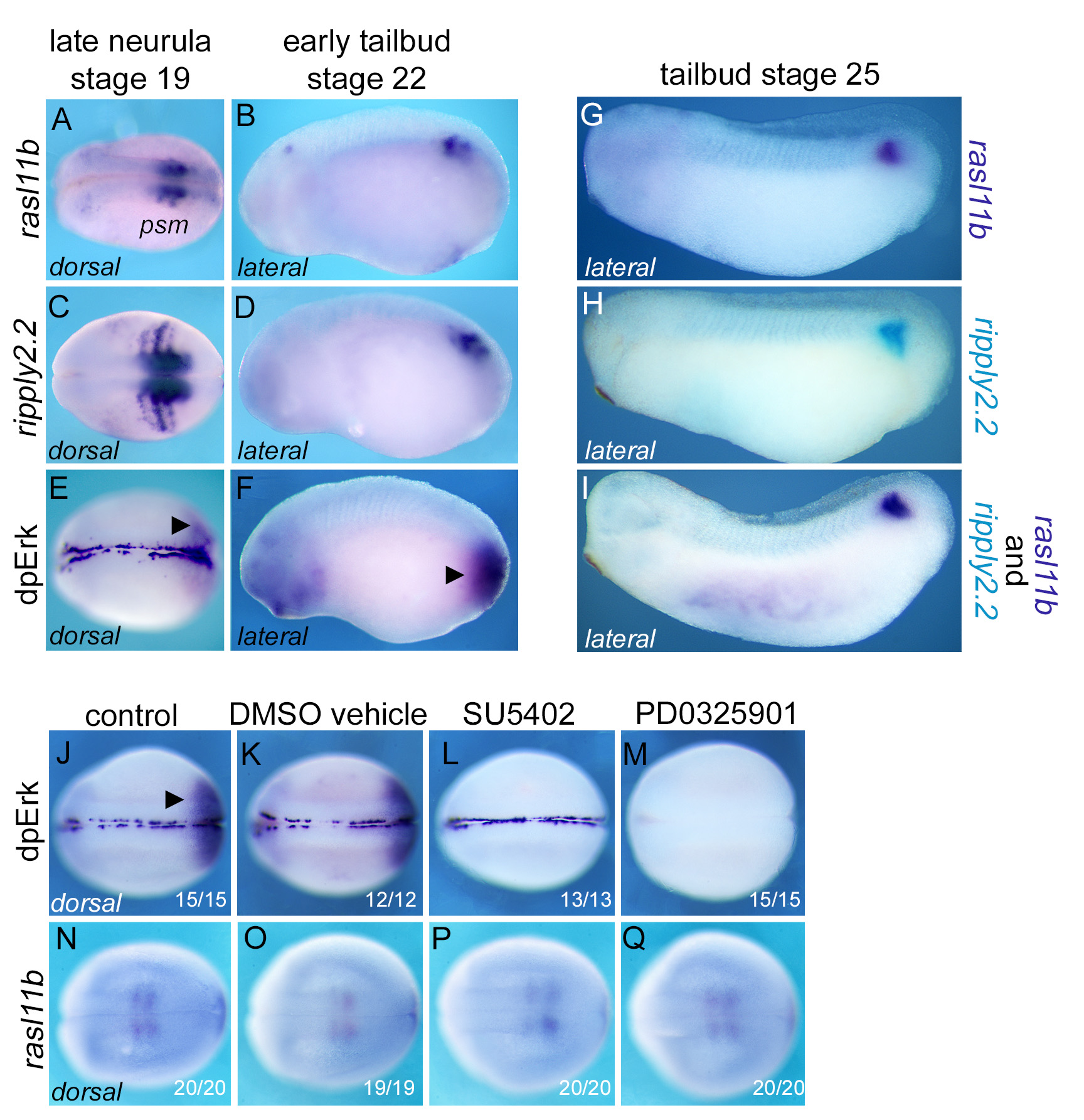

Supplement: S7 Fig — A and B, in situ hybridization of rasl11b expression at late neurula stage 19 and tailbud stage 22. C and D expression of ripply2.2 at late neurula stage 19 and tailbud stage 22. E and F localisation of dpErk by immunohistochemistry at stage 19 and 22. G-I in situ hybridization analysis of rasl11b (magenta) and ripply2.2 (cyan) expression in the pre-somitic mesoderm at mid-tailbud stage 25. J-M, neurula stage embryos showing immunolocalisation of dpErk and, N-Q, in situ localisation of rasl11b expression in untreated control embryos and embryos treated with 0.1% DMSO vehicle, 200 μM Fgfr inhibitor SU5402 or 25 μM Mek inhibitor PD0325901. Black arrow indicates posterior mesoderm. NB Indicated n values are from a representative experiment. (JPG) [file pone.0286040.s007.jpg]

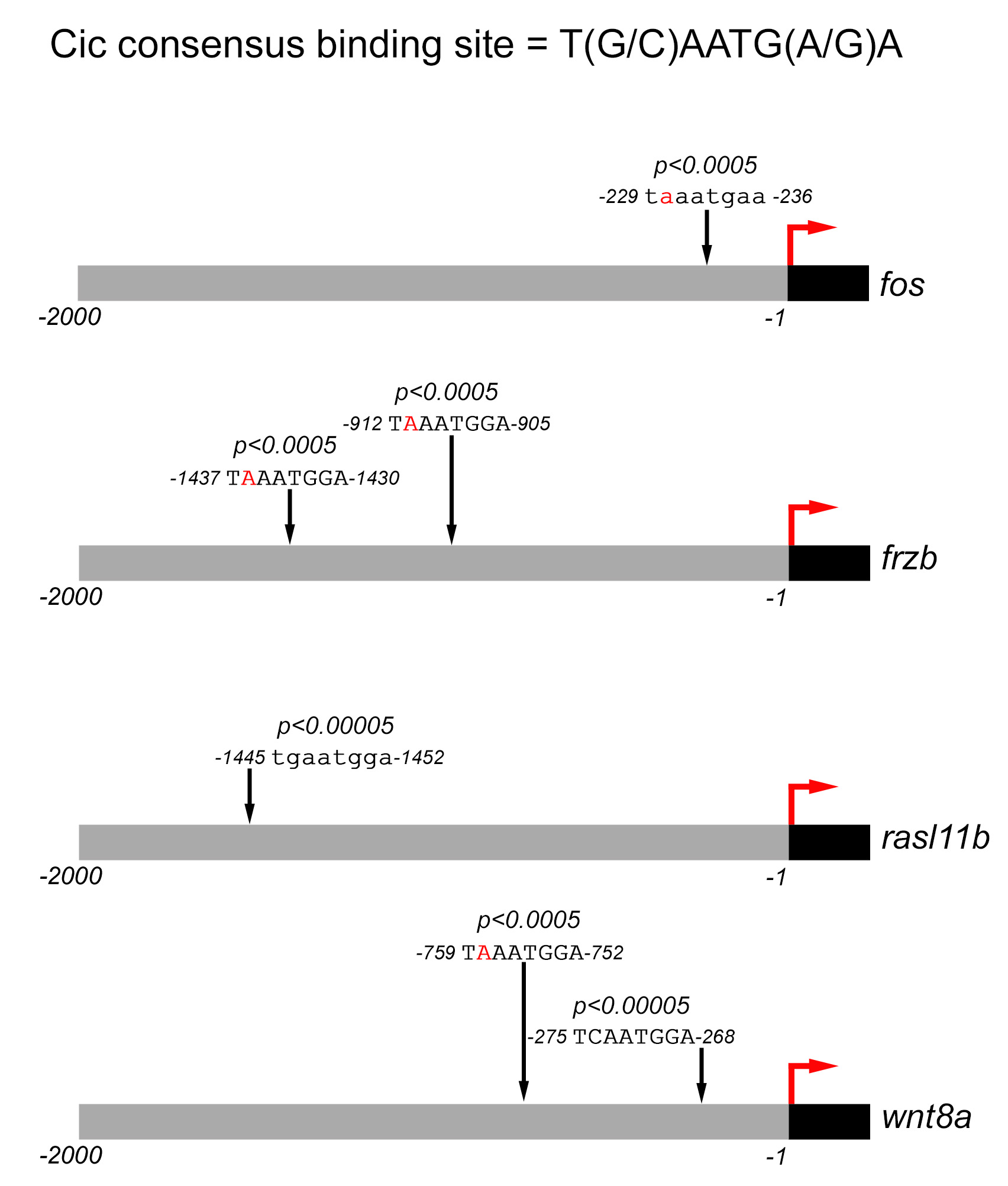

Supplement: S8 Fig — Position, orientation and statistical significance of Cic consensus binding sites in the 2kb upstream regions of putative targets of Cic regulation in Xenopus, identified by scanning with the FIMO tool from the MEME suite. Lower-case and upper-case sites are on the plus and minus strands, respectively. (JPG) [file pone.0286040.s008.jpg]

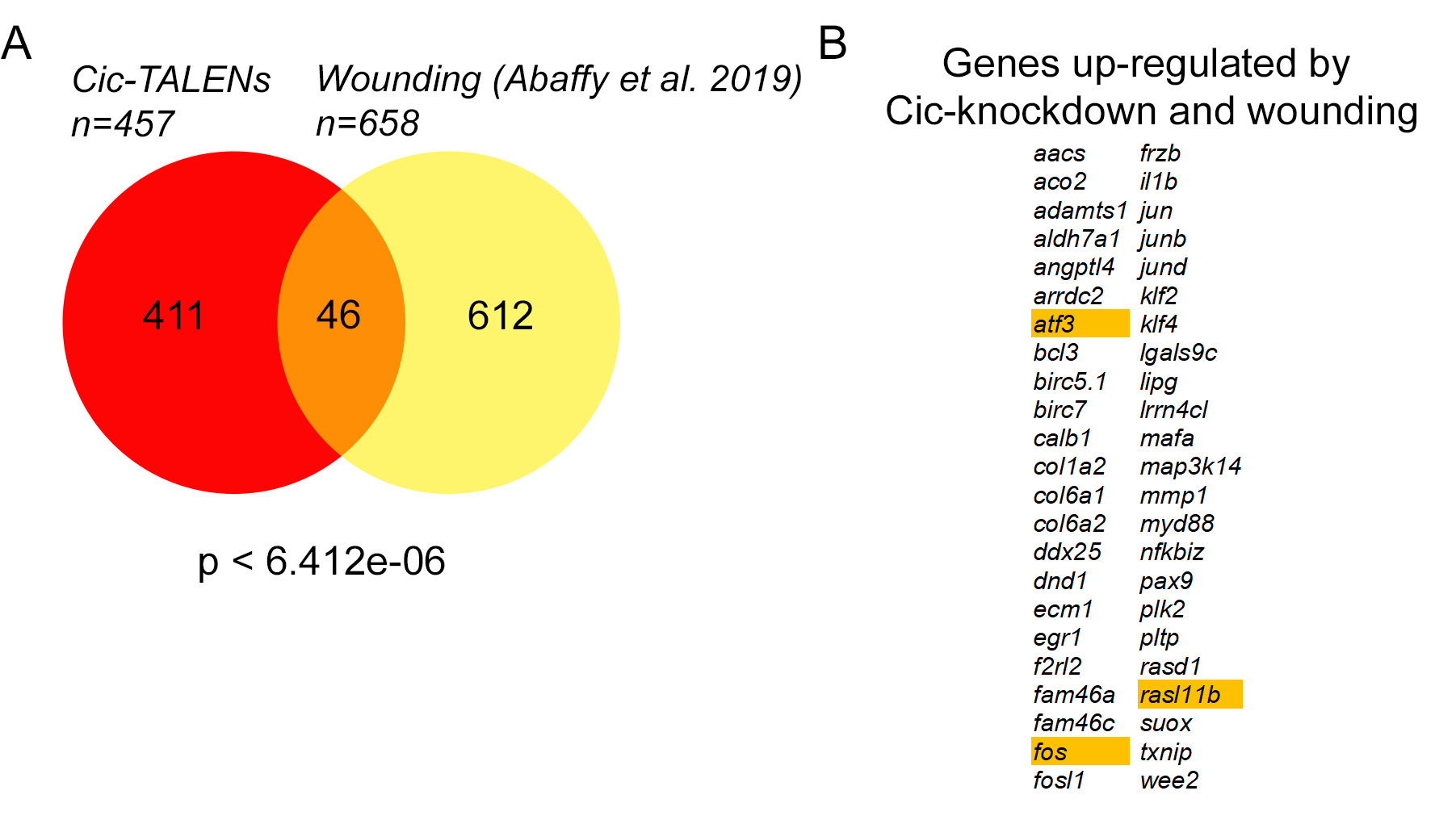

Supplement: S9 Fig — Xenopus laevis wounding datasets from [50] were filtered to only include genes annotated in the Xenopus tropicalis genome (12992 genes). Datasets for 30–90 minutes post-wounding were combined and filtered according to the following criteria, fold up-regulation ≥1.75 and p-adj ≤0.1. Duplicate gene entries were removed from the Xenopus laevis dataset. A, shows the highly significant overlap of genes up-regulated by Cic-knockdown in Xenopus tropicalis and wounding in Xenopus laevis. B, Genes up-regulated by Cic-knockdown and ectodermal wounding. Orange shading indicates genes analysed at the site of wounding in this study (Fig 5). (JPG) [file pone.0286040.s009.jpg]

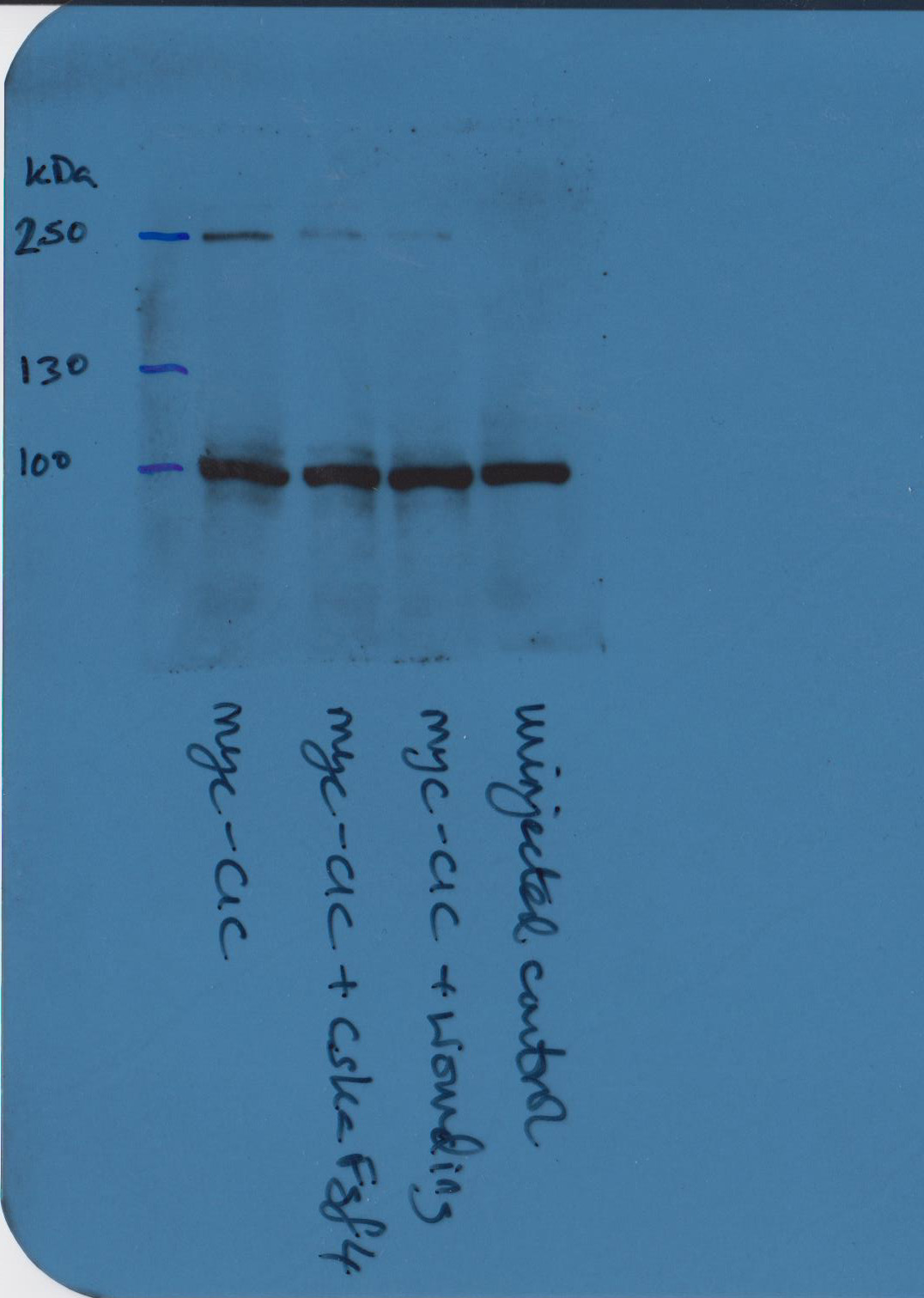

Supplement: S10 Fig — (JPG) [file pone.0286040.s010.jpg]

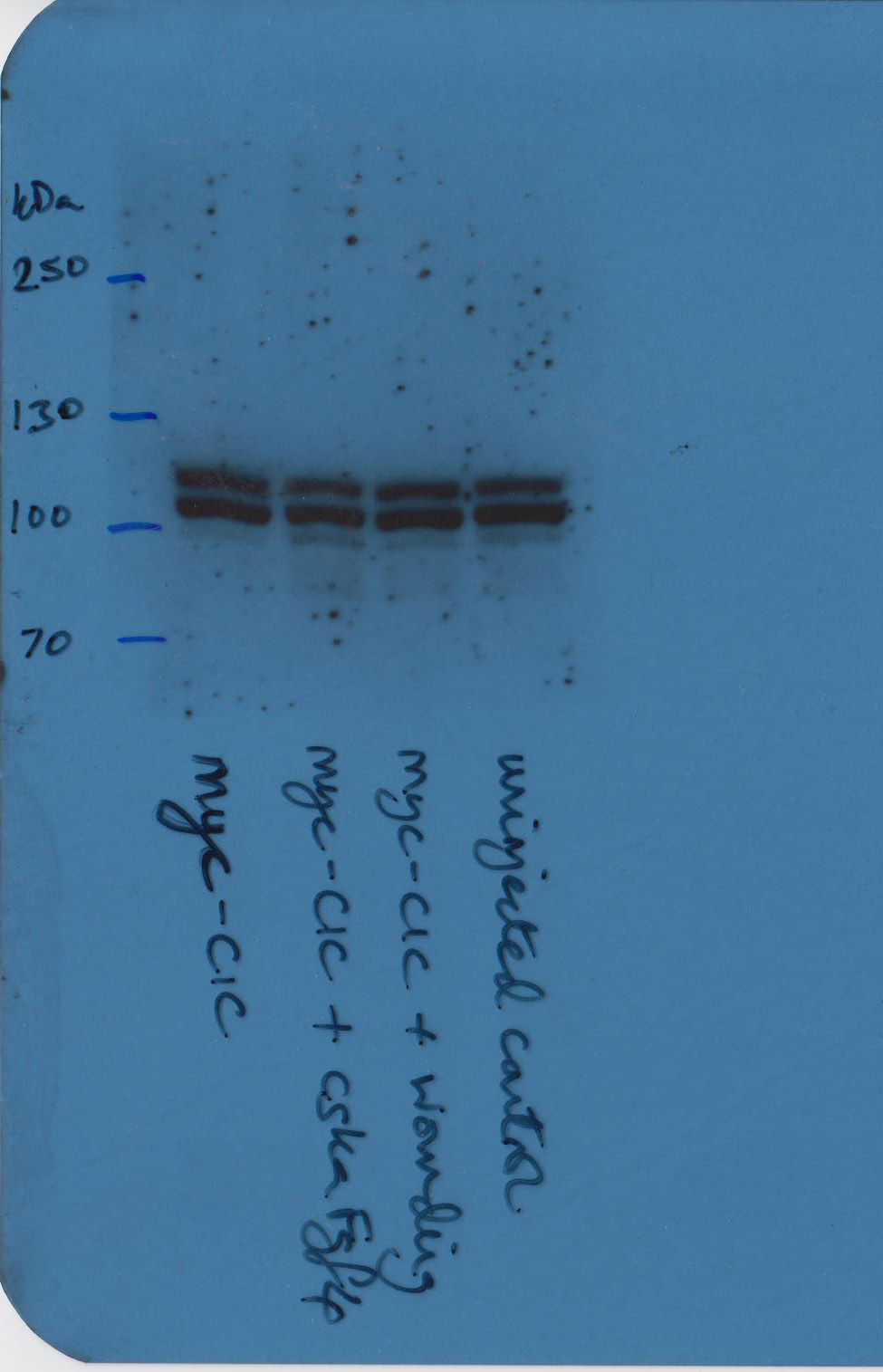

Supplement: S11 Fig — (JPG) [file pone.0286040.s011.jpg]
