# Supplementary material for: Regulation of gene expression downstream of a novel Fgf/Erk pathway during Xenopus development
Source: PLoS One. 2023 Oct 19;18(10):e0286040. doi: 10.1371/journal.pone.0286040 (PMC10586617; doi:10.1371/journal.pone.0286040)
Supplement: S2 Table — (DOCX) [file pone.0286040.s013.docx]

**Table_S6 PANTHER gene ontology slim molecular function analysis** **of genes up-regulated by Cic knockdown** (enrichment ≥2, FDR≤0.05)

| **PANTHER GO-Slim Molecular Function** | **Xenopus tropicalis - REFLIST (22504)** | **Input (1125)** | **Input (expected)** | **Input**  **(fold Enrichment)** | **Input**  **(False Discovery Rate)** |
| --- | --- | --- | --- | --- | --- |
| fibroblast growth factor binding (GO:0017134) | 6 | 4 | 0.3 | 13.34 | 1.78E-02 |
| mRNA 3'-UTR binding (GO:0003730) | 34 | 10 | 1.7 | 5.88 | 8.39E-04 |
| growth factor binding (GO:0019838) | 29 | 7 | 1.45 | 4.83 | 2.72E-02 |
| DNA-binding transcription factor activity, RNA polymerase II-specific (GO:0000981) | 873 | 112 | 43.64 | 2.57 | 4.01E-16 |
| RNA polymerase II cis-regulatory region sequence-specific DNA binding (GO:0000978) | 799 | 100 | 39.94 | 2.5 | 8.84E-14 |
| RNA polymerase II transcription regulatory region sequence-specific DNA binding (GO:0000977) | 970 | 121 | 48.49 | 2.5 | 3.29E-16 |
| cis-regulatory region sequence-specific DNA binding (GO:0000987) | 813 | 100 | 40.64 | 2.46 | 1.78E-13 |
| sequence-specific DNA binding (GO:0043565) | 1064 | 130 | 53.19 | 2.44 | 2.97E-16 |
| sequence-specific double-stranded DNA binding (GO:1990837) | 1037 | 125 | 51.84 | 2.41 | 3.98E-16 |
| transcription cis-regulatory region binding (GO:0000976) | 1017 | 122 | 50.84 | 2.4 | 1.73E-15 |
| transcription regulatory region nucleic acid binding (GO:0001067) | 1017 | 122 | 50.84 | 2.4 | 1.58E-15 |
| DNA-binding transcription factor activity (GO:0003700) | 969 | 116 | 48.44 | 2.39 | 7.77E-15 |
| double-stranded DNA binding (GO:0003690) | 1084 | 129 | 54.19 | 2.38 | 3.73E-16 |
| transcription regulator activity (GO:0140110) | 1150 | 135 | 57.49 | 2.35 | 2.81E-16 |
| mRNA binding (GO:0003729) | 190 | 22 | 9.5 | 2.32 | 1.25E-02 |
| DNA binding (GO:0003677) | 1264 | 144 | 63.19 | 2.28 | 2.89E-16 |
| nucleic acid binding (GO:0003676) | 1841 | 184 | 92.03 | 2 | 3.54E-16 |

**Highlight key**

| Terms associated with growth factor binding |  |
| --- | --- |
| Terms associated with DNA binding and transcriptional regulation |  |
