# Supplementary material for: Regulation of gene expression downstream of a novel Fgf/Erk pathway during Xenopus development
Source: PLoS One. 2023 Oct 19;18(10):e0286040. doi: 10.1371/journal.pone.0286040 (PMC10586617; doi:10.1371/journal.pone.0286040)
Supplement: S3 Table — (DOCX) [file pone.0286040.s014.docx]

**Table_S7 PANTHER gene ontology biological process analysis of genes up-regulated by Cic knockdown** (enrichment ≥2, FDR≤0.05)

| **PANTHER GO-Slim Biological Process** | **Xenopus tropicalis - REFLIST (22504)** | **Input (1125)** | **Input (expected)** | **Input**  **(fold Enrichment)** | **Input**  **(False Discovery rate)** |
| --- | --- | --- | --- | --- | --- |
| smoothened signaling pathway (GO:0007224) | 16 | 6 | 0.8 | 7.5 | 1.25E-02 |
| cellular process involved in reproduction in multicellular organism (GO:0022412) | 25 | 7 | 1.25 | 5.6 | 1.72E-02 |
| gamete generation (GO:0007276) | 37 | 8 | 1.85 | 4.33 | 2.72E-02 |
| multicellular organismal reproductive process (GO:0048609) | 41 | 8 | 2.05 | 3.9 | 4.35E-02 |
| multicellular organism reproduction (GO:0032504) | 41 | 8 | 2.05 | 3.9 | 4.31E-02 |
| head development (GO:0060322) | 55 | 10 | 2.75 | 3.64 | 2.39E-02 |
| brain development (GO:0007420) | 55 | 10 | 2.75 | 3.64 | 2.36E-02 |
| cell fate commitment (GO:0045165) | 65 | 11 | 3.25 | 3.39 | 2.31E-02 |
| negative regulation of translation (GO:0017148) | 60 | 10 | 3 | 3.33 | 3.72E-02 |
| negative regulation of cellular amide metabolic process (GO:0034249) | 62 | 10 | 3.1 | 3.23 | 4.36E-02 |
| central nervous system development (GO:0007417) | 103 | 16 | 5.15 | 3.11 | 5.60E-03 |
| regulation of plasma membrane bounded cell projection organization (GO:0120035) | 82 | 12 | 4.1 | 2.93 | 3.73E-02 |
| regulation of cell projection organization (GO:0031344) | 83 | 12 | 4.15 | 2.89 | 3.93E-02 |
| canonical Wnt signaling pathway (GO:0060070) | 92 | 13 | 4.6 | 2.83 | 3.27E-02 |
| Wnt signaling pathway (GO:0016055) | 101 | 14 | 5.05 | 2.77 | 2.75E-02 |
| cell-cell signaling by wnt (GO:0198738) | 102 | 14 | 5.1 | 2.75 | 2.86E-02 |
| cell surface receptor signaling pathway involved in cell-cell signaling (GO:1905114) | 113 | 15 | 5.65 | 2.66 | 2.73E-02 |
| negative regulation of gene expression (GO:0010629) | 163 | 21 | 8.15 | 2.58 | 9.25E-03 |
| posttranscriptional regulation of gene expression (GO:0010608) | 132 | 17 | 6.6 | 2.58 | 1.94E-02 |
| transmembrane receptor protein tyrosine kinase signaling pathway (GO:0007169) | 182 | 23 | 9.1 | 2.53 | 5.58E-03 |
| cell differentiation (GO:0030154) | 761 | 85 | 38.04 | 2.23 | 4.21E-09 |
| cellular developmental process (GO:0048869) | 765 | 85 | 38.24 | 2.22 | 4.57E-09 |
| regulation of gene expression (GO:0010468) | 1881 | 204 | 94.03 | 2.17 | 2.13E-21 |
| negative regulation of macromolecule biosynthetic process (GO:0010558) | 268 | 29 | 13.4 | 2.16 | 8.65E-03 |
| negative regulation of cellular macromolecule biosynthetic process (GO:2000113) | 268 | 29 | 13.4 | 2.16 | 8.52E-03 |
| regulation of transcription, DNA-templated (GO:0006355) | 1550 | 166 | 77.49 | 2.14 | 6.47E-17 |
| regulation of nucleic acid-templated transcription (GO:1903506) | 1550 | 166 | 77.49 | 2.14 | 6.20E-17 |
| regulation of RNA biosynthetic process (GO:2001141) | 1550 | 166 | 77.49 | 2.14 | 5.96E-17 |
| regulation of transcription by RNA polymerase II (GO:0006357) | 1292 | 138 | 64.59 | 2.14 | 5.03E-14 |
| negative regulation of biosynthetic process (GO:0009890) | 273 | 29 | 13.65 | 2.12 | 9.65E-03 |
| negative regulation of cellular biosynthetic process (GO:0031327) | 273 | 29 | 13.65 | 2.12 | 9.52E-03 |
| regulation of cellular macromolecule biosynthetic process (GO:2000112) | 1678 | 178 | 83.89 | 2.12 | 1.03E-17 |
| regulation of macromolecule biosynthetic process (GO:0010556) | 1680 | 178 | 83.99 | 2.12 | 1.05E-17 |
| regulation of RNA metabolic process (GO:0051252) | 1674 | 177 | 83.69 | 2.12 | 1.85E-17 |
| RNA biosynthetic process (GO:0032774) | 1620 | 171 | 80.99 | 2.11 | 5.88E-17 |
| regulation of cellular biosynthetic process (GO:0031326) | 1697 | 179 | 84.83 | 2.11 | 1.31E-17 |
| nucleic acid-templated transcription (GO:0097659) | 1613 | 170 | 80.64 | 2.11 | 7.72E-17 |
| transcription, DNA-templated (GO:0006351) | 1613 | 170 | 80.64 | 2.11 | 7.46E-17 |
| regulation of biosynthetic process (GO:0009889) | 1703 | 179 | 85.13 | 2.1 | 1.55E-17 |
| regulation of nucleobase-containing compound metabolic process (GO:0019219) | 1731 | 181 | 86.53 | 2.09 | 1.53E-17 |
| transcription by RNA polymerase II (GO:0006366) | 1335 | 138 | 66.74 | 2.07 | 5.59E-13 |
| neuron differentiation (GO:0030182) | 330 | 34 | 16.5 | 2.06 | 8.21E-03 |
| regulation of primary metabolic process (GO:0080090) | 2147 | 221 | 107.33 | 2.06 | 5.27E-21 |
| regulation of metabolic process (GO:0019222) | 2373 | 244 | 118.63 | 2.06 | 8.23E-23 |
| regulation of macromolecule metabolic process (GO:0060255) | 2255 | 231 | 112.73 | 2.05 | 1.16E-21 |
| regulation of cellular metabolic process (GO:0031323) | 2221 | 227 | 111.03 | 2.04 | 3.64E-21 |
| enzyme linked receptor protein signaling pathway (GO:0007167) | 284 | 29 | 14.2 | 2.04 | 1.79E-02 |
| regulation of nitrogen compound metabolic process (GO:0051171) | 2111 | 215 | 105.53 | 2.04 | 8.76E-20 |
| aromatic compound biosynthetic process (GO:0019438) | 1874 | 189 | 93.68 | 2.02 | 5.89E-17 |
| heterocycle biosynthetic process (GO:0018130) | 1871 | 188 | 93.53 | 2.01 | 9.17E-17 |
| nucleobase-containing compound biosynthetic process (GO:0034654) | 1843 | 185 | 92.13 | 2.01 | 1.56E-16 |
| organic cyclic compound biosynthetic process (GO:1901362) | 1906 | 191 | 95.28 | 2 | 7.42E-17 |

**Highlight key**

| Terms associated with signalling |  |
| --- | --- |
| Terms associated with gene transcription |  |
| Terms associated with neuronal and head development |  |
