# Supplementary material for: Regulation of gene expression downstream of a novel Fgf/Erk pathway during Xenopus development
Source: PLoS One. 2023 Oct 19;18(10):e0286040. doi: 10.1371/journal.pone.0286040 (PMC10586617; doi:10.1371/journal.pone.0286040)
Supplement: S4 Table — (DOCX) [file pone.0286040.s015.docx]

**Table_S8** **Genes significantly up-regulated by Fgf4 overexpression and Cic knockdown** (p≤0.01 and effect size ≥1.75)

| Gene | Control mean | Cska-Fgf4  mean | Cic-TALEN mean | Fgf4/Cont pval | Fgf4/Cont effect size | Cic-TALEN/Cont pval | Cic-TALEN/Cont effect size |
| --- | --- | --- | --- | --- | --- | --- | --- |
| adamts1 | 8.30 | 32.53 | 36.02 | 9.90E-03 | 2.33 | 1.38E-03 | 2.85 |
| adcy4 | 1.49 | 4.71 | 3.88 | 2.69E-05 | 2.44 | 4.54E-04 | 2.10 |
| apold1 | 0.68 | 7.03 | 4.60 | 2.38E-03 | 5.45 | 5.61E-03 | 4.69 |
| arhgap11a.2 | 2.10 | 5.76 | 11.35 | 1.28E-03 | 1.87 | 3.55E-09 | 3.15 |
| arrdc2 | 5.56 | 20.81 | 19.05 | 2.74E-11 | 2.50 | 6.79E-10 | 2.34 |
| atf3 | 4.58 | 10.92 | 12.37 | 9.34E-04 | 1.76 | 7.74E-05 | 1.96 |
| azin2 | 35.20 | 209.32 | 237.35 | 1.51E-04 | 3.43 | 8.88E-05 | 3.58 |
| bcl11a | 0.72 | 2.15 | 1.86 | 4.42E-03 | 2.30 | 6.90E-03 | 2.21 |
| bri3 | 4.56 | 12.04 | 11.20 | 2.05E-03 | 1.86 | 2.52E-03 | 1.84 |
| c4bpa | 5.29 | 18.67 | 16.03 | 1.47E-03 | 2.20 | 7.15E-03 | 1.95 |
| cbx4 | 2.71 | 11.87 | 8.24 | 6.23E-07 | 2.87 | 1.69E-04 | 2.22 |
| cpa6 | 0.99 | 3.18 | 14.38 | 6.03E-03 | 2.36 | 2.94E-09 | 6.39 |
| dscaml1 | 13.58 | 25.65 | 57.38 | 7.05E-03 | 1.88 | 2.71E-07 | 3.35 |
| egr1 | 2.96 | 31.29 | 10.42 | 1.39E-05 | 4.28 | 9.78E-03 | 2.37 |
| fam83c | 10.49 | 34.14 | 29.54 | 1.54E-08 | 2.08 | 2.65E-06 | 1.84 |
| fgd3 | 0.52 | 3.70 | 4.49 | 3.77E-09 | 3.97 | 9.83E-11 | 4.54 |
| fos | 3.61 | 39.43 | 29.93 | 3.15E-24 | 5.38 | 2.66E-19 | 4.43 |
| fosl1 | 0.93 | 4.45 | 4.35 | 3.77E-03 | 3.00 | 2.89E-03 | 3.09 |
| fpgt | 0.56 | 1.85 | 1.85 | 5.01E-03 | 2.59 | 3.93E-03 | 2.66 |
| frzb | 12.67 | 78.04 | 122.32 | 2.83E-04 | 3.21 | 2.88E-06 | 4.49 |
| galr3 | 0.81 | 2.88 | 3.16 | 5.26E-03 | 2.37 | 6.42E-03 | 2.33 |
| ier3 | 58.55 | 200.36 | 225.42 | 4.60E-05 | 2.33 | 3.29E-06 | 2.63 |
| insm2 | 0.65 | 1.83 | 4.46 | 1.42E-03 | 2.04 | 1.17E-08 | 3.57 |
| jun | 14.77 | 40.56 | 54.70 | 6.77E-03 | 2.06 | 2.11E-05 | 3.11 |
| lgals9c | 12.75 | 40.66 | 100.54 | 3.30E-04 | 2.01 | 3.84E-10 | 3.38 |
| LOC100485132 | 0.44 | 1.80 | 1.90 | 4.20E-03 | 2.25 | 4.05E-04 | 2.72 |
| LOC100486038 | 7.09 | 24.92 | 19.20 | 2.12E-05 | 2.66 | 4.67E-04 | 2.24 |
| LOC100493036 | 1.14 | 18.02 | 32.16 | 2.36E-03 | 15.04 | 7.38E-04 | 20.26 |
| LOC100493666 | 1.46 | 4.46 | 4.03 | 2.05E-03 | 2.03 | 3.21E-03 | 1.97 |
| LOC100495743 | 48.08 | 117.05 | 96.15 | 1.66E-04 | 2.08 | 3.57E-03 | 1.76 |
| LOC100496651 | 0.16 | 4.30 | 11.23 | 8.72E-03 | 4.75 | 2.33E-06 | 16.52 |
| LOC100498550 | 0.83 | 5.16 | 5.46 | 6.62E-03 | 3.14 | 2.87E-03 | 3.51 |
| LOC101730746 | 1.75 | 4.92 | 4.09 | 5.78E-05 | 1.99 | 5.92E-04 | 1.80 |
| LOC101730897 | 1.27 | 2.97 | 3.42 | 2.69E-03 | 1.90 | 7.73E-04 | 2.05 |
| LOC101731310 | 3.25 | 8.55 | 19.34 | 5.39E-03 | 2.90 | 3.05E-05 | 4.93 |
| LOC101731765 | 15.65 | 52.02 | 31.28 | 2.74E-07 | 2.51 | 1.48E-03 | 1.76 |
| LOC101732940 | 0.55 | 2.87 | 4.58 | 1.09E-03 | 3.24 | 2.18E-05 | 4.61 |
| LOC101733948 | 7.72 | 33.21 | 22.08 | 4.34E-05 | 2.68 | 9.45E-04 | 2.22 |
| LOC101734677 | 0.77 | 2.57 | 3.35 | 4.93E-03 | 2.22 | 4.75E-04 | 2.70 |
| LOC101734729 | 0.62 | 2.96 | 1.86 | 1.22E-04 | 2.79 | 3.98E-03 | 2.16 |
| LOC105945272 | 0.45 | 2.11 | 1.77 | 8.25E-04 | 3.44 | 1.44E-03 | 3.24 |
| LOC105945708 | 1.19 | 7.07 | 3.43 | 1.19E-07 | 2.75 | 4.08E-04 | 1.96 |
| LOC105945972 | 0.88 | 3.45 | 4.12 | 6.90E-03 | 2.60 | 2.34E-03 | 2.93 |
| LOC105947461 | 29.18 | 90.79 | 66.35 | 1.42E-04 | 2.23 | 5.22E-03 | 1.80 |
| LOC105947813 | 0.26 | 2.12 | 2.39 | 4.86E-08 | 4.95 | 2.60E-08 | 5.11 |
| LOC108647658 | 0.50 | 2.42 | 2.39 | 2.19E-03 | 2.89 | 3.54E-03 | 2.75 |
| LOC733556 | 2.72 | 8.17 | 22.78 | 2.13E-03 | 2.20 | 2.54E-09 | 4.62 |
| mixl1 | 0.73 | 13.88 | 14.67 | 6.12E-03 | 5.86 | 5.14E-04 | 9.40 |
| mmp1 | 0.42 | 5.26 | 10.28 | 7.67E-05 | 4.65 | 6.36E-08 | 8.19 |
| mmrn2 | 0.85 | 7.23 | 5.82 | 7.41E-05 | 3.62 | 7.33E-04 | 2.99 |
| nfkbiz | 5.09 | 14.61 | 15.15 | 5.28E-05 | 2.02 | 1.39E-05 | 2.13 |
| oxct1 | 1.23 | 3.65 | 2.92 | 1.31E-03 | 2.09 | 6.18E-03 | 1.87 |
| rab20 | 1.55 | 3.61 | 4.43 | 5.54E-03 | 1.77 | 1.92E-03 | 1.90 |
| rab7b | 0.64 | 2.41 | 6.35 | 5.06E-03 | 2.53 | 2.54E-07 | 5.49 |
| rasl11b | 19.00 | 51.67 | 77.70 | 1.12E-06 | 1.99 | 1.35E-11 | 2.60 |
| rgl2 | 0.86 | 3.54 | 14.35 | 2.11E-06 | 2.62 | 1.78E-20 | 6.59 |
| sgk1 | 82.21 | 317.57 | 290.16 | 5.66E-08 | 2.66 | 9.23E-07 | 2.42 |
| smpdl3a | 0.28 | 1.87 | 1.81 | 2.58E-04 | 3.16 | 6.92E-04 | 2.91 |
| tmem128 | 0.66 | 1.84 | 2.12 | 8.56E-03 | 1.95 | 5.50E-03 | 2.03 |
| tnfrsf10b | 3.22 | 7.84 | 25.41 | 4.20E-04 | 1.92 | 3.79E-16 | 4.52 |
| trex2 | 2.71 | 13.65 | 50.11 | 1.98E-03 | 3.08 | 6.40E-08 | 7.14 |
| trim2 | 0.87 | 2.91 | 2.82 | 3.83E-04 | 2.15 | 6.71E-04 | 2.09 |
| txnip | 13.09 | 28.95 | 40.80 | 3.53E-03 | 1.81 | 3.14E-05 | 2.33 |
| usp2 | 1.65 | 5.30 | 13.08 | 1.01E-03 | 2.17 | 4.37E-08 | 3.64 |
| wnt8a | 67.94 | 210.55 | 162.54 | 8.58E-06 | 2.20 | 6.24E-04 | 1.83 |
