# Supplementary material for: Regulation of gene expression downstream of a novel Fgf/Erk pathway during Xenopus development
Source: PLoS One. 2023 Oct 19;18(10):e0286040. doi: 10.1371/journal.pone.0286040 (PMC10586617; doi:10.1371/journal.pone.0286040)
Supplement: S5 Table — (DOCX) [file pone.0286040.s016.docx]

**Table_S9** **Function of genes significantly up-regulated by Fgf4 overexpression and Cic knockdown**

| Gene symbol | Gene name | Annotation |
| --- | --- | --- |
| [adamts1](http://www.ncbi.nlm.nih.gov/entrez/query.fcgi?db=Gene&cmd=search&term=adamts1) | ADAM metallopeptidase with thrombospondin type 1 motif 1 | Negative regulator of Fgf signalling (Suga et al., 2006) |
| [adcy4](http://www.ncbi.nlm.nih.gov/entrez/query.fcgi?db=Gene&cmd=search&term=adcy4) | adenylate cyclase 4 |  |
| [apold1](http://www.ncbi.nlm.nih.gov/entrez/query.fcgi?db=Gene&cmd=search&term=apold1) | apolipoprotein L domain containing 1 | Early response gene involved in endothelial cell signalling (Regard et al., 2004) |
| [arhgap11a.2](http://www.ncbi.nlm.nih.gov/entrez/query.fcgi?db=Gene&cmd=search&term=arhgap11a.2) | Rho GTPase activating protein 11A, gene 2 | Proto-oncogene (Lawson and Der, 2018) |
| [arrdc2](http://www.ncbi.nlm.nih.gov/entrez/query.fcgi?db=Gene&cmd=search&term=arrdc2) | arrestin domain containing 2 |  |
| [atf3](http://www.ncbi.nlm.nih.gov/entrez/query.fcgi?db=Gene&cmd=search&term=atf3) | activating transcription factor 3 | Transcription factor involved in metabolic regulation and cancer (Ku and Cheng, 2020) |
| [azin2](http://www.ncbi.nlm.nih.gov/entrez/query.fcgi?db=Gene&cmd=search&term=azin2) | antizyme inhibitor 2 |  |
| [bcl11a](http://www.ncbi.nlm.nih.gov/entrez/query.fcgi?db=Gene&cmd=search&term=bcl11a) | BCL11A, BAF complex component | Regulators of polyamine metabolism (López-Contreras et al., 2010) |
| [bri3](http://www.ncbi.nlm.nih.gov/entrez/query.fcgi?db=Gene&cmd=search&term=bri3) | brain protein I3 | Integral membrane protein involved in neuronal differentiation (Gong et al., 2008) |
| [c4bpa](http://www.ncbi.nlm.nih.gov/entrez/query.fcgi?db=Gene&cmd=search&term=c4bpa) | complement component 4 binding protein, alpha |  |
| [cbx4](http://www.ncbi.nlm.nih.gov/entrez/query.fcgi?db=Gene&cmd=search&term=cbx4) | chromobox 4 | Epigenetic regulation via binding to H3K27me3 (van Wijnen et al., 2021) |
| [cpa6](http://www.ncbi.nlm.nih.gov/entrez/query.fcgi?db=Gene&cmd=search&term=cpa6) | carboxypeptidase A6 | Proteolytic processing of enzymes. |
| [dscaml1](http://www.ncbi.nlm.nih.gov/entrez/query.fcgi?db=Gene&cmd=search&term=dscaml1) | down syndrome cell adhesion molecule like 1 | Ig-like transmembrane protein involved in neural development (Montesinos, 2014) |
| [egr1](http://www.ncbi.nlm.nih.gov/entrez/query.fcgi?db=Gene&cmd=search&term=egr1) | early growth response 1 | Transcription factor regulated by Fgf/Map kinase signalling (Branney et al., 2009; Nentwich et al., 2009) |
| [fam83c](http://www.ncbi.nlm.nih.gov/entrez/query.fcgi?db=Gene&cmd=search&term=fam83c) | family with sequence similarity 83 member C | FAM83 proteins are oncogenic and positive regulators of map kinase signalling (Bartel et al., 2016) |
| [fgd3](http://www.ncbi.nlm.nih.gov/entrez/query.fcgi?db=Gene&cmd=search&term=fgd3) | FYVE, RhoGEF and PH domain containing 3 |  |
| [fos](http://www.ncbi.nlm.nih.gov/entrez/query.fcgi?db=Gene&cmd=search&term=fos) | Fos proto-oncogene | AP-1 transcription factor subunit. AP-1 is involved in mediating Fgf signalling in Xenopus (Dong et al., 1996; Kim et al., 1998) |
| [fosl1](http://www.ncbi.nlm.nih.gov/entrez/query.fcgi?db=Gene&cmd=search&term=fosl1) | Fos-like antigen 1 | AP-1 transcription factor subunit |
| [fpgt](http://www.ncbi.nlm.nih.gov/entrez/query.fcgi?db=Gene&cmd=search&term=fpgt) | fucose-1-phosphate guanylyltransferase |  |
| [frzb](http://www.ncbi.nlm.nih.gov/entrez/query.fcgi?db=Gene&cmd=search&term=frzb) | frizzled related protein | Secreted wnt antagonist (Wang et al., 1997). Transcriptional target of Fgf signalling(Branney et al., 2009) |
| [galr3](http://www.ncbi.nlm.nih.gov/entrez/query.fcgi?db=Gene&cmd=search&term=galr3) | galanin receptor 3 | Neuropeptide receptor |
| [ier3](http://www.ncbi.nlm.nih.gov/entrez/query.fcgi?db=Gene&cmd=search&term=ier3) | immediate early response 3 | Activated by multiple growth factor pathways, including map kinase/erk, pi3 kinase and nfkb (Arlt and Schäfer, 2011) |
| [insm2](http://www.ncbi.nlm.nih.gov/entrez/query.fcgi?db=Gene&cmd=search&term=insm2) | insulinoma-associated 2 |  |
| [jun](http://www.ncbi.nlm.nih.gov/entrez/query.fcgi?db=Gene&cmd=search&term=jun) | jun proto-oncogene | AP-1 transcription factor subunit. AP-1 is involved in mediating Fgf signalling in Xenopus (Dong et al., 1996; Kim et al., 1998) |
| [lgals9c](http://www.ncbi.nlm.nih.gov/entrez/query.fcgi?db=Gene&cmd=search&term=lgals9c) | lectin, galactoside-binding, soluble, 9C |  |
| [LOC100485132](http://www.ncbi.nlm.nih.gov/entrez/query.fcgi?db=Gene&cmd=search&term=LOC100485132) |  | TRPM8 channel-associated factor homolog |
| [LOC100486038](http://www.ncbi.nlm.nih.gov/entrez/query.fcgi?db=Gene&cmd=search&term=LOC100486038) |  | uncharacterized protein coding locus LOC100486038 |
| [LOC100493036](http://www.ncbi.nlm.nih.gov/entrez/query.fcgi?db=Gene&cmd=search&term=LOC100493036) |  | provisional histone H3 homolog |
| [LOC100493666](http://www.ncbi.nlm.nih.gov/entrez/query.fcgi?db=Gene&cmd=search&term=LOC100493666) |  | uncharacterized protein coding locus LOC100493666 |
| [LOC100495743](http://www.ncbi.nlm.nih.gov/entrez/query.fcgi?db=Gene&cmd=search&term=LOC100495743) |  | uncharacterized protein coding locus LOC100495743 |
| [LOC100496651](http://www.ncbi.nlm.nih.gov/entrez/query.fcgi?db=Gene&cmd=search&term=LOC100496651) |  | provisional homeobox100496651 |
| [LOC100498550](http://www.ncbi.nlm.nih.gov/entrez/query.fcgi?db=Gene&cmd=search&term=LOC100498550) |  | provisional pleckstrin homology domain-containing family N member 1 |
| [LOC101730746](http://www.ncbi.nlm.nih.gov/entrez/query.fcgi?db=Gene&cmd=search&term=LOC101730746) |  | uncharacterised locus LOC101730746 |
| [LOC101730897](http://www.ncbi.nlm.nih.gov/entrez/query.fcgi?db=Gene&cmd=search&term=LOC101730897) |  | provisional leishmanolysin like peptidase 2 |
| [LOC101731310](http://www.ncbi.nlm.nih.gov/entrez/query.fcgi?db=Gene&cmd=search&term=LOC101731310) |  | uncharacterized protein coding locus LOC101731310 |
| [LOC101731765](http://www.ncbi.nlm.nih.gov/entrez/query.fcgi?db=Gene&cmd=search&term=LOC101731765) |  | uncharacterized protein coding locus LOC101731765 |
| [LOC101732940](http://www.ncbi.nlm.nih.gov/entrez/query.fcgi?db=Gene&cmd=search&term=LOC101732940) |  | provisional interleukin-12 receptor subunit beta-2 |
| [LOC101733948](http://www.ncbi.nlm.nih.gov/entrez/query.fcgi?db=Gene&cmd=search&term=LOC101733948) |  | provisional coiled-coil domain-containing protein 77 |
| [LOC101734677](http://www.ncbi.nlm.nih.gov/entrez/query.fcgi?db=Gene&cmd=search&term=LOC101734677) |  | uncharacterized protein coding locus LOC101734677 |
| [LOC101734729](http://www.ncbi.nlm.nih.gov/entrez/query.fcgi?db=Gene&cmd=search&term=LOC101734729) |  | provisional von Willebrand factor C and EGF domain-containing protein-like |
| [LOC105945272](http://www.ncbi.nlm.nih.gov/entrez/query.fcgi?db=Gene&cmd=search&term=LOC105945272) |  | uncharacterized protein coding locus LOC105945272 |
| [LOC105945708](http://www.ncbi.nlm.nih.gov/entrez/query.fcgi?db=Gene&cmd=search&term=LOC105945708) |  | provisional olfactory receptor 5B21-like |
| [LOC105945972](http://www.ncbi.nlm.nih.gov/entrez/query.fcgi?db=Gene&cmd=search&term=LOC105945972) |  | uncharacterized non-coding RNA locus LOC105945972 |
| [LOC105947461](http://www.ncbi.nlm.nih.gov/entrez/query.fcgi?db=Gene&cmd=search&term=LOC105947461) |  | uncharacterized protein coding locus LOC100495743 |
| [LOC105947813](http://www.ncbi.nlm.nih.gov/entrez/query.fcgi?db=Gene&cmd=search&term=LOC105947813) |  | uncharacterized protein coding locus LOC105947813 |
| [LOC108647658](http://www.ncbi.nlm.nih.gov/entrez/query.fcgi?db=Gene&cmd=search&term=LOC108647658) |  | uncharacterized non-coding RNA locus LOC108647658 |
| [LOC733556](http://www.ncbi.nlm.nih.gov/entrez/query.fcgi?db=Gene&cmd=search&term=LOC733556) |  | uncharacterized gene XB5734924 |
| [mixl1](http://www.ncbi.nlm.nih.gov/entrez/query.fcgi?db=Gene&cmd=search&term=mixl1) | mix paired homeobox | Regulator of germ layer specification and Fgf signalling (Colas et al., 2008; Pereira et al., 2012) |
| [mmp1](http://www.ncbi.nlm.nih.gov/entrez/query.fcgi?db=Gene&cmd=search&term=mmp1) | matrix metallopeptidase 1 | Known target of map kinase/erk signalling (Park et al., 2011) |
| [mmrn2](http://www.ncbi.nlm.nih.gov/entrez/query.fcgi?db=Gene&cmd=search&term=mmrn2) | multimerin 2 | Large homopolymeric protein |
| [nfkbiz](http://www.ncbi.nlm.nih.gov/entrez/query.fcgi?db=Gene&cmd=search&term=nfkbiz) | NFKB inhibitor zeta | Inducible inhibitor of the nfkb transcription factor (Muta, 2006) |
| [oxct1](http://www.ncbi.nlm.nih.gov/entrez/query.fcgi?db=Gene&cmd=search&term=oxct1) | 3-oxoacid CoA-transferase 1 | Involved in cancer cell metabolism (Zhang and Xie, 2017) |
| [rab20](http://www.ncbi.nlm.nih.gov/entrez/query.fcgi?db=Gene&cmd=search&term=rab20) | RAB20, member RAS oncogene family | Ras family GTPase involved in membrane trafficking and EGFR degradation (Pei et al., 2015) |
| [rab7b](http://www.ncbi.nlm.nih.gov/entrez/query.fcgi?db=Gene&cmd=search&term=rab7b) | RAB7B, member RAS oncogene family | Ras family GTPase involved in membrane trafficking (Distefano et al., 2015) |
| [rasl11b](http://www.ncbi.nlm.nih.gov/entrez/query.fcgi?db=Gene&cmd=search&term=rasl11b) | RAS like family 11 member B | Atypical member of the ras family of GTPases lacking membrane localisation signal. Implicated as an agonist of crypto/frl1 signalling (Pézeron et al., 2008) |
| [rgl2](http://www.ncbi.nlm.nih.gov/entrez/query.fcgi?db=Gene&cmd=search&term=rgl2) | ral guanine nucleotide dissociation stimulator like 2 | Ras interacting protein involved in regulating map kinase pi3 kinase(Scotland et al., 2013) pathways (Fischer et al., 2003) |
| [sgk1](http://www.ncbi.nlm.nih.gov/entrez/query.fcgi?db=Gene&cmd=search&term=sgk1) | serum/glucocorticoid regulated kinase 1 | Serine/threonine kinase required for normal axis development in Xenopus (Endo et al., 2011) |
| [smpdl3a](http://www.ncbi.nlm.nih.gov/entrez/query.fcgi?db=Gene&cmd=search&term=smpdl3a) | sphingomyelin phosphodiesterase acid-like 3A |  |
| [tmem128](http://www.ncbi.nlm.nih.gov/entrez/query.fcgi?db=Gene&cmd=search&term=tmem128) | transmembrane protein 128 |  |
| [tnfrsf10b](http://www.ncbi.nlm.nih.gov/entrez/query.fcgi?db=Gene&cmd=search&term=tnfrsf10b) | tumor necrosis factor receptor superfamily member 10b | Transduction of apoptotic signals |
| [trex2](http://www.ncbi.nlm.nih.gov/entrez/query.fcgi?db=Gene&cmd=search&term=trex2) | three prime repair exonuclease 2 | Regulator of mRNA export and gene expression (García-Oliver et al., 2012) |
| [trim2](http://www.ncbi.nlm.nih.gov/entrez/query.fcgi?db=Gene&cmd=search&term=trim2) | tripartite motif containing 2 | E3 ubiquitin ligase involved in neurogenesis (Lokapally et al., 2020) |
| [txnip](http://www.ncbi.nlm.nih.gov/entrez/query.fcgi?db=Gene&cmd=search&term=txnip) | thioredoxin interacting protein | Involved in stress responses and neurodegenerative disease (Tsubaki et al., 2020) |
| [usp2](http://www.ncbi.nlm.nih.gov/entrez/query.fcgi?db=Gene&cmd=search&term=usp2) | ubiquitin specific peptidase 2 | Deubiquinating enzyme expressed at high levels in some cancer (Kitamura and Hashimoto, 2021) |
| [wnt8a](http://www.ncbi.nlm.nih.gov/entrez/query.fcgi?db=Gene&cmd=search&term=wnt8a) | wnt family member 8A | Transcriptional target of Fgf signalling (Branney et al., 2009) |

**References**

Arlt, A., Schäfer, H., 2011. Role of the immediate early response 3 (IER3) gene in cellular stress response, inflammation and tumorigenesis. Eur. J. Cell Biol. 90, 545–552.

Bartel, C.A., Parameswaran, N., Cipriano, R., Jackson, M.W., 2016. FAM83 proteins: Fostering new interactions to drive oncogenic signaling and therapeutic resistance. Oncotarget 7, 52597–52612.

Branney, P. a., Faas, L., Steane, S.E., Pownall, M.E., Isaacs, H.V., 2009. Characterisation of the fibroblast growth factor dependent transcriptome in early development. PLoS One 4, e4951–e4951.

Cao, Y., Zhao, H., Hollemann, T., Chen, Y., Grunz, H., 2001. Tissue-specific expression of an Ornithine decarboxylase paralogue, XODC2, in Xenopus laevis. Mech. Dev. 102, 243–6.

Colas, A., Cartry, J., Buisson, I., Umbhauer, M., Smith, J.C., Riou, J.-F., 2008. Mix.1/2-dependent control of FGF availability during gastrulation is essential for pronephros development in Xenopus. Dev. Biol. 320, 351–365.

Distefano, M.B., Kjos, I., Bakke, O., Progida, C., 2015. Rab7b at the intersection of intracellular trafficking and cell migration. Commun. Integr. Biol. 8, e1023492.

Dong, Z., Xu, R.H., Kim, J., Zhan, S.N., Ma, W.Y., Colburn, N.H., Kung, H., 1996. AP-1/jun is required for early Xenopus development and mediates mesoderm induction by fibroblast growth factor but not by activin. J. Biol. Chem. 271, 9942–9946.

Endo, T., Kusakabe, M., Sunadome, K., Yamamoto, T., Nishida, E., 2011. The kinase SGK1 in the endoderm and mesoderm promotes ectodermal survival by down-regulating components of the death-inducing signaling complex. Sci. Signal. 4, ra2.

Fischer, T.H., Brittain, J., Trabalzini, L., Banes, A.J., White, G.C., Smith, C.J., Nichols, T.C., 2003. The ras-binding domain of ral GDS-like protein-2 as a ras inhibitor in smooth muscle cells. Biochem. Biophys. Res. Commun. 305, 934–940.

García-Oliver, E., García-Molinero, V., Rodríguez-Navarro, S., 2012. mRNA export and gene expression: the SAGA-TREX-2 connection. Biochim. Biophys. Acta 1819, 555–565.

Gong, Y., Wu, J., Qiang, H., Liu, B., Chi, Z., Chen, T., Yin, B., Peng, X., Yuan, J., 2008. BRI3 associates with SCG10 and attenuates NGF-induced neurite outgrowth in PC12 cells. BMB Rep. 41, 287–293.

Kim, J., Lin, J.J., Xu, R.H., Kung, H.F., 1998. Mesoderm induction by heterodimeric AP-1 (c-Jun and c-Fos) and its involvement in mesoderm formation through the embryonic fibroblast growth factor/Xbra autocatalytic loop during the early development of Xenopus embryos. J. Biol. Chem. 273, 1542–1550.

Kitamura, H., Hashimoto, M., 2021. USP2-Related Cellular Signaling and Consequent Pathophysiological Outcomes. Int. J. Mol. Sci. 22. https://doi.org/10.3390/ijms22031209

Ku, H.-C., Cheng, C.-F., 2020. Master Regulator Activating Transcription Factor 3 (ATF3) in Metabolic Homeostasis and Cancer. Front. Endocrinol. 11, 556.

Lawson, C.D., Der, C.J., 2018. Filling GAPs in our knowledge: ARHGAP11A and RACGAP1 act as oncogenes in basal-like breast cancers. Small GTPases 9, 290–296.

Lokapally, A., Neuhaus, H., Herfurth, J., Hollemann, T., 2020. Interplay of TRIM2 E3 Ubiquitin Ligase and ALIX/ESCRT Complex: Control of Developmental Plasticity During Early Neurogenesis. Cells 9. https://doi.org/10.3390/cells9071734

López-Contreras, A.J., Ramos-Molina, B., Cremades, A., Peñafiel, R., 2010. Antizyme inhibitor 2: molecular, cellular and physiological aspects. Amino Acids 38, 603–611.

Montesinos, M.L., 2014. Roles for DSCAM and DSCAML1 in central nervous system development and disease. Adv Neurobiol 8, 249–270.

Muta, T., 2006. IkappaB-zeta: an inducible regulator of nuclear factor-kappaB. Vitam. Horm. 74, 301–316.

Nentwich, O., Dingwell, K.S., Nordheim, A., Smith, J.C., 2009. Downstream of FGF during mesoderm formation in Xenopus: the roles of Elk-1 and Egr-1. Dev. Biol. 336, 313–326.

Park, S., Jung, H.H., Park, Y.H., Ahn, J.S., Im, Y.-H., 2011. ERK/MAPK pathways play critical roles in EGFR ligands-induced MMP1 expression. Biochem. Biophys. Res. Commun. 407, 680–686.

Pei, G., Schnettger, L., Bronietzki, M., Repnik, U., Griffiths, G., Gutierrez, M.G., 2015. Interferon-γ-inducible Rab20 regulates endosomal morphology and EGFR degradation in macrophages. Mol. Biol. Cell 26, 3061–3070.

Pereira, L.A., Wong, M.S., Mei Lim, S., Stanley, E.G., Elefanty, A.G., 2012. The Mix family of homeobox genes--key regulators of mesendoderm formation during vertebrate development. Dev. Biol. 367, 163–177.

Pézeron, G., Lambert, G., Dickmeis, T., Strähle, U., Rosa, F.M., Mourrain, P., 2008. Rasl11b knock down in zebrafish suppresses one-eyed-pinhead mutant phenotype. PLoS One 3, e1434.

Regard, J.B., Scheek, S., Borbiev, T., Lanahan, A.A., Schneider, A., Demetriades, A.-M., Hiemisch, H., Barnes, C.A., Verin, A.D., Worley, P.F., 2004. Verge: a novel vascular early response gene. J. Neurosci. 24, 4092–4103.

Scotland, R.L., Allen, L., Hennings, L.J., Post, G.R., Post, S.R., 2013. The ral exchange factor rgl2 promotes cardiomyocyte survival and inhibits cardiac fibrosis. PLoS One 8, e73599.

Suga, A., Hikasa, H., Taira, M., 2006. Xenopus ADAMTS1 negatively modulates FGF signaling independent of its metalloprotease activity. Dev. Biol. 295, 26–39.

Tsubaki, H., Tooyama, I., Walker, D.G., 2020. Thioredoxin-Interacting Protein (TXNIP) with Focus on Brain and Neurodegenerative Diseases. Int. J. Mol. Sci. 21. https://doi.org/10.3390/ijms21249357

van Wijnen, A.J., Bagheri, L., Badreldin, A.A., Larson, A.N., Dudakovic, A., Thaler, R., Paradise, C.R., Wu, Z., 2021. Biological functions of chromobox (CBX) proteins in stem cell self-renewal, lineage-commitment, cancer and development. Bone 143, 115659.

Wang, S., Krinks, M., Lin, K., Luyten, F.P., Moos M, Jr., 1997. Frzb, a secreted protein expressed in the Spemann organizer, binds and inhibits Wnt-8. Cell 88, 757–766.

Zhang, S., Xie, C., 2017. The role of OXCT1 in the pathogenesis of cancer as a rate-limiting enzyme of ketone body metabolism. Life Sci. 183, 110–115.
