# Supplementary material for: Regulation of gene expression downstream of a novel Fgf/Erk pathway during Xenopus development
Source: PLoS One. 2023 Oct 19;18(10):e0286040. doi: 10.1371/journal.pone.0286040 (PMC10586617; doi:10.1371/journal.pone.0286040)
Supplement: S6 Table — (DOCX) [file pone.0286040.s017.docx]

**Table_S11** **Genes significantly down-regulated by Fgf4 overexpression and Cic knockdown** (p≤0.01 and effect size≤0.57)

| Gene | Control mean | Cska-Fgf4  mean | Cic-TALEN mean | Fgf4/Cont pval | Fgf4/Cont effect size | Cic-TALEN/Cont pval | Cic-TALEN/Cont effect size |
| --- | --- | --- | --- | --- | --- | --- | --- |
| bmpr1b | 36.72 | 14.60 | 14.02 | 3.53E-03 | 0.48 | 6.44E-03 | 0.51 |
| cebpa | 23.57 | 3.47 | 4.93 | 1.07E-07 | 0.28 | 2.73E-05 | 0.36 |
| cnga2 | 4.00 | 0.86 | 1.27 | 4.55E-04 | 0.32 | 4.51E-03 | 0.40 |
| cygb | 75.44 | 27.18 | 31.71 | 5.12E-06 | 0.48 | 1.56E-04 | 0.55 |
| dmrta1 | 8.74 | 2.27 | 2.33 | 2.10E-03 | 0.41 | 1.35E-03 | 0.39 |
| draxin | 11.88 | 5.62 | 4.18 | 4.79E-03 | 0.56 | 1.07E-04 | 0.45 |
| fezf1 | 8.56 | 2.24 | 1.68 | 1.45E-03 | 0.36 | 4.69E-04 | 0.32 |
| fezf2 | 12.21 | 4.23 | 3.15 | 8.98E-03 | 0.33 | 2.04E-03 | 0.27 |
| foxg1 | 15.00 | 2.15 | 1.54 | 2.43E-03 | 0.25 | 3.92E-04 | 0.20 |
| gfi1 | 9.24 | 0.91 | 1.61 | 3.20E-05 | 0.19 | 5.13E-03 | 0.33 |
| LOC100487395 | 12.51 | 4.59 | 4.62 | 2.08E-03 | 0.52 | 1.02E-03 | 0.50 |
| LOC100494953 | 2.53 | 0.53 | 0.79 | 1.93E-05 | 0.25 | 1.27E-03 | 0.35 |
| LOC101731177 | 2.05 | 0.78 | 0.68 | 8.22E-03 | 0.46 | 5.38E-03 | 0.44 |
| nes | 15.55 | 6.09 | 6.13 | 4.61E-03 | 0.53 | 1.94E-03 | 0.50 |
| nfasc | 3.01 | 1.19 | 1.03 | 8.07E-03 | 0.52 | 4.00E-03 | 0.49 |
| nkain1 | 10.34 | 3.85 | 4.13 | 1.71E-04 | 0.55 | 2.50E-04 | 0.55 |
| nova2 | 37.74 | 14.69 | 14.22 | 4.35E-03 | 0.54 | 1.49E-03 | 0.50 |
| nr2f2 | 24.01 | 6.79 | 6.37 | 5.72E-03 | 0.40 | 4.43E-03 | 0.39 |
| pax6 | 82.11 | 16.77 | 16.09 | 8.96E-06 | 0.28 | 5.30E-05 | 0.31 |
| pdp2 | 26.76 | 5.86 | 7.66 | 4.65E-04 | 0.41 | 1.11E-03 | 0.43 |
| pitx1 | 23.53 | 4.13 | 5.51 | 1.57E-03 | 0.24 | 8.65E-03 | 0.30 |
| pitx2 | 38.52 | 11.41 | 9.05 | 3.03E-03 | 0.38 | 7.02E-04 | 0.33 |
| pou2f3 | 12.01 | 2.37 | 3.84 | 4.48E-05 | 0.30 | 8.58E-03 | 0.47 |
| pou3f2 | 6.01 | 1.91 | 1.32 | 5.99E-03 | 0.48 | 2.26E-04 | 0.37 |
| rasgef1a | 12.30 | 4.03 | 4.16 | 4.27E-04 | 0.46 | 4.68E-04 | 0.47 |
| rax | 55.81 | 5.94 | 5.70 | 5.09E-03 | 0.16 | 2.44E-03 | 0.13 |
| sfrp2 | 85.05 | 35.22 | 32.18 | 4.27E-03 | 0.56 | 1.14E-03 | 0.52 |
| six3 | 32.91 | 7.01 | 6.64 | 3.26E-03 | 0.25 | 1.41E-03 | 0.22 |
| slc23a2 | 56.74 | 19.95 | 26.55 | 1.16E-03 | 0.49 | 7.79E-03 | 0.56 |
| slc7a2.1 | 11.39 | 4.47 | 3.71 | 3.14E-04 | 0.47 | 2.50E-04 | 0.46 |
| spam1 | 10.32 | 0.85 | 1.14 | 1.52E-03 | 0.12 | 4.93E-03 | 0.16 |
| spib | 30.44 | 2.09 | 3.39 | 2.06E-07 | 0.14 | 1.08E-05 | 0.18 |
| tmem119 | 7.63 | 1.83 | 1.22 | 2.75E-03 | 0.31 | 1.71E-03 | 0.29 |
| unc13d | 3.81 | 0.58 | 0.34 | 1.26E-03 | 0.27 | 9.90E-05 | 0.21 |
| wnt8b | 5.20 | 1.46 | 1.90 | 1.56E-03 | 0.45 | 4.55E-03 | 0.49 |
