# Supplementary material for: Regulation of gene expression downstream of a novel Fgf/Erk pathway during Xenopus development
Source: PLoS One. 2023 Oct 19;18(10):e0286040. doi: 10.1371/journal.pone.0286040 (PMC10586617; doi:10.1371/journal.pone.0286040)
Supplement: S7 Table — (DOCX) [file pone.0286040.s018.docx]

**Table_S12** **Function of genes significantly down-regulated by Fgf4 overexpression and Cic knockdown**

| Gene symbol | Gene name | Annotation |
| --- | --- | --- |
| [bmpr1b](http://www.ncbi.nlm.nih.gov/entrez/query.fcgi?db=Gene&cmd=search&term=bmpr1b) | bone morphogenetic protein receptor type 1B | serine/threonine kinase receptor involved in mesoderm patterning (Maeno et al., 1994) |
| [cebpa](http://www.ncbi.nlm.nih.gov/entrez/query.fcgi?db=Gene&cmd=search&term=cebpa) | CCAAT enhancer binding protein alpha | marker of myeloid lineage (Costa et al., 2008) |
| [cnga2](http://www.ncbi.nlm.nih.gov/entrez/query.fcgi?db=Gene&cmd=search&term=cnga2) | cyclic nucleotide gated channel subunit alpha 2 | Involved in olfaction |
| [cygb](http://www.ncbi.nlm.nih.gov/entrez/query.fcgi?db=Gene&cmd=search&term=cygb) | cytoglobin | Globin family gene (Nakade et al., 2015) |
| [dmrta1](http://www.ncbi.nlm.nih.gov/entrez/query.fcgi?db=Gene&cmd=search&term=dmrta1) | DMRT like family A1 | Dmrt family transcription factor expressed in anterior nervous system (Huang et al., 2005) |
| [draxin](http://www.ncbi.nlm.nih.gov/entrez/query.fcgi?db=Gene&cmd=search&term=draxin) | dorsal inhibitory axon guidance protein | Wnt signalling antagonist involved in neural crest migration (Hutchins and Bronner, 2018) |
| [fezf1](http://www.ncbi.nlm.nih.gov/entrez/query.fcgi?db=Gene&cmd=search&term=fezf1) | FEZ family zinc finger 1 | zinc finger protein involved in anterior neural patterning (Rodríguez-Seguel et al., 2009) |
| [fezf2](http://www.ncbi.nlm.nih.gov/entrez/query.fcgi?db=Gene&cmd=search&term=fezf2) | FEZ family zinc finger 1 | zinc finger protein involved in anterior neural patterning (Rodríguez-Seguel et al., 2009) |
| [foxg1](http://www.ncbi.nlm.nih.gov/entrez/query.fcgi?db=Gene&cmd=search&term=foxg1) | forkhead box G1 | Transcription factor involved in anterior neural development (Hardcastle and Papalopulu, 2000) |
| [gfi1](http://www.ncbi.nlm.nih.gov/entrez/query.fcgi?db=Gene&cmd=search&term=gfi1) | growth factor independent 1 transcriptional repressor | Transcription factor expressed in blood islands and placode (Ciau-Uitz et al., 2010) |
| [LOC100487395](http://www.ncbi.nlm.nih.gov/entrez/query.fcgi?db=Gene&cmd=search&term=LOC100487395) |  | provisional fidgetin like2 |
| [LOC100494953](http://www.ncbi.nlm.nih.gov/entrez/query.fcgi?db=Gene&cmd=search&term=LOC100494953) |  | provisional neural-cadherin |
| [LOC101731177](http://www.ncbi.nlm.nih.gov/entrez/query.fcgi?db=Gene&cmd=search&term=LOC101731177) |  | provisional zinc finger protein OZF |
| [nes](http://www.ncbi.nlm.nih.gov/entrez/query.fcgi?db=Gene&cmd=search&term=nes) | nestin | neuronal intermediate filament protein (Hemmati-Brivanlou et al., 1992) |
| [nfasc](http://www.ncbi.nlm.nih.gov/entrez/query.fcgi?db=Gene&cmd=search&term=nfasc) | neurofascin | neural cell adhesion molecule |
| [nkain1](http://www.ncbi.nlm.nih.gov/entrez/query.fcgi?db=Gene&cmd=search&term=nkain1) | sodium/potassium transporting ATPase interacting 1 | predicted membrane protein |
| [nova2](http://www.ncbi.nlm.nih.gov/entrez/query.fcgi?db=Gene&cmd=search&term=nova2) | NOVA alternative splicing regulator 2 | regulator of alternative splicing in neural tissue (Jelen et al., 2007) |
| [nr2f2](http://www.ncbi.nlm.nih.gov/entrez/query.fcgi?db=Gene&cmd=search&term=nr2f2) | nuclear receptor subfamily 2 group F member 2 | steroid hormone receptor expressed in anterior nervous system (van der Wees et al., 1996) |
| [pax6](http://www.ncbi.nlm.nih.gov/entrez/query.fcgi?db=Gene&cmd=search&term=pax6) | paired box 6 | homeodomain transcription factor in eye and neural development (Hirsch and Harris, 1997) |
| [pdp2](http://www.ncbi.nlm.nih.gov/entrez/query.fcgi?db=Gene&cmd=search&term=pdp2) | pyruvate dehyrogenase phosphatase catalytic subunit 2 | Mitochondrial enzyme |
| [pitx1](http://www.ncbi.nlm.nih.gov/entrez/query.fcgi?db=Gene&cmd=search&term=pitx1) | paired like homeodomain 1 | transcription factor involved in anterior development (Hollemann and Pieler, 1999) |
| [pitx2](http://www.ncbi.nlm.nih.gov/entrez/query.fcgi?db=Gene&cmd=search&term=pitx2) | paired like homeodomain 2 | transcription factor involved in anterior development and left-right asymmetry (Ryan et al., 1998) |
| [pou2f3](http://www.ncbi.nlm.nih.gov/entrez/query.fcgi?db=Gene&cmd=search&term=pou2f3) | POU class 2 homeobox 3 | POU family homeodomain transcription factor |
| [pou3f2](http://www.ncbi.nlm.nih.gov/entrez/query.fcgi?db=Gene&cmd=search&term=pou3f2) | POU class 3 homeobox 2 | POU family homeodomain transcription factor expressed in anterior nervous system (Cosse-Etchepare et al., 2018) |
| [rasgef1a](http://www.ncbi.nlm.nih.gov/entrez/query.fcgi?db=Gene&cmd=search&term=rasgef1a) | rasGEF domain family member 1A | guanine nucleotide exchange factor |
| [rax](http://www.ncbi.nlm.nih.gov/entrez/query.fcgi?db=Gene&cmd=search&term=rax) | retina and anterior neural fold homeobox | transcription factor expressed in anterior nervous system and involved in retinal development (Andreazzoli et al., 1999) |
| [sfrp2](http://www.ncbi.nlm.nih.gov/entrez/query.fcgi?db=Gene&cmd=search&term=sfrp2) | secreted frizzled related protein 2 | secreted Wnt antagonist expressed in anterior ectoderm (Takahashi et al., 2005) |
| [six3](http://www.ncbi.nlm.nih.gov/entrez/query.fcgi?db=Gene&cmd=search&term=six3) | six homeobox 3 | transcription factor involved in anterior neural development (Gestri et al., 2005) |
| [slc23a2](http://www.ncbi.nlm.nih.gov/entrez/query.fcgi?db=Gene&cmd=search&term=slc23a2) | solute carrier family 23 member 2 | ascorbic acid transporter |
| [slc7a2.1](http://www.ncbi.nlm.nih.gov/entrez/query.fcgi?db=Gene&cmd=search&term=slc7a2.1) | solute carrier family 7 member 2, gene 1 | cationic amino acid transporter |
| [spam1](http://www.ncbi.nlm.nih.gov/entrez/query.fcgi?db=Gene&cmd=search&term=spam1) | sperm adhesion molecule 1 | cell adhesion molecule expressed expression in ventral region of embryo |
| [spib](http://www.ncbi.nlm.nih.gov/entrez/query.fcgi?db=Gene&cmd=search&term=spib) | spi-B transcription factor | ETS family transcription factor involved in myeloid development (Costa et al., 2008) |
| [tmem119](http://www.ncbi.nlm.nih.gov/entrez/query.fcgi?db=Gene&cmd=search&term=tmem119) | transmembrane protein 119 | Expressed in microglia in mammals |
| [unc13d](http://www.ncbi.nlm.nih.gov/entrez/query.fcgi?db=Gene&cmd=search&term=unc13d) | unc-13 homolog D |  |
| [wnt8b](http://www.ncbi.nlm.nih.gov/entrez/query.fcgi?db=Gene&cmd=search&term=wnt8b) | Wnt family member 8B | Wnt family ligand expressed in anterior neural plate (Merzdorf and Sive, 2006) |

References

Ciau-Uitz, A., Pinheiro, P., Gupta, R., Enver, T., Patient, R., 2010. Tel1/ETV6 specifies blood stem cells through the agency of VEGF signaling. Dev. Cell 18, 569–578.

Costa, R.M.B., Soto, X., Chen, Y., Zorn, A.M., Amaya, E., 2008. spib is required for primitive myeloid development in Xenopus. Blood 112, 2287–2296.

Hardcastle, Z., Papalopulu, N., 2000. Distinct effects of XBF-1 in regulating the cell cycle inhibitor p27(XIC1) and imparting a neural fate. Development 127, 1303–1314.

Hemmati-Brivanlou, A., Mann, R.W., Harland, R.M., 1992. A protein expressed in the growth cones of embryonic vertebrate neurons defines a new class of intermediate filament protein. Neuron 9, 417–428.

Hirsch, N., Harris, W.A., 1997. Xenopus Pax-6 and retinal development. J. Neurobiol. 32, 45–61.

Hollemann, T., Pieler, T., 1999. Xpitx-1: a homeobox gene expressed during pituitary and cement gland formation of Xenopus embryos. Mech. Dev. 88, 249–252.

Huang, X., Hong, C.-S., O’Donnell, M., Saint-Jeannet, J.-P., 2005. The doublesex-related gene, XDmrt4, is required for neurogenesis in the olfactory system. Proc. Natl. Acad. Sci. U. S. A. 102, 11349–11354.

Hutchins, E.J., Bronner, M.E., 2018. Draxin acts as a molecular rheostat of canonical Wnt signaling to control cranial neural crest EMT. J. Cell Biol. 217, 3683–3697.

Jelen, N., Ule, J., Zivin, M., Darnell, R.B., 2007. Evolution of Nova-dependent splicing regulation in the brain. PLoS Genet. 3, 1838–1847.

Maeno, M., Ong, R.C., Suzuki, A., Ueno, N., Kung, H.F., 1994. A truncated bone morphogenetic protein 4 receptor alters the fate of ventral mesoderm to dorsal mesoderm: Roles of animal pole tissue in the development of ventral mesoderm. Proc. Natl. Acad. Sci. U. S. A. 91, 10260–10264.

Rodríguez-Seguel, E., Alarcón, P., Gómez-Skarmeta, J.L., 2009. The Xenopus Irx genes are essential for neural patterning and define the border between prethalamus and thalamus through mutual antagonism with the anterior repressors Fezf and Arx. Dev. Biol. 329, 258–268.

Ryan, A.K., Blumberg, B., Rodriguez-Esteban, C., Yonei-Tamura, S., Tamura, K., Tsukui, T., de la Peña, J., Sabbagh, W., Greenwald, J., Choe, S., Norris, D.P., Robertson, E.J., Evans, R.M., Rosenfeld, M.G., Izpisúa Belmonte, J.C., 1998. Pitx2 determines left-right asymmetry of internal organs in vertebrates. Nature 394, 545–551.

van der Wees, J., Matharu, P.J., de Roos, K., Destrée, O.H., Godsave, S.F., Durston, A.J., Sweeney, G.E., 1996. Developmental expression and differential regulation by retinoic acid of Xenopus COUP-TF-A and COUP-TF-B. Mech. Dev. 54, 173–184.

Andreazzoli, M., Gestri, G., Angeloni, D., Menna, E., Barsacchi, G., 1999. Role of Xrx1 in Xenopus eye and anterior brain development. Development 126, 2451-2460.

Cosse-Etchepare, C., Gervi, I., Buisson, I., Formery, L., Schubert, M., Riou, J.F., Umbhauer, M., Le Bouffant, R., 2018. Pou3f transcription factor expression during embryonic development highlights distinct pou3f3 and pou3f4 localization in the Xenopus laevis kidney. Int J Dev Biol 62, 325-333.

Gestri, G., Carl, M., Appolloni, I., Wilson, S.W., Barsacchi, G., Andreazzoli, M., 2005. Six3 functions in anterior neural plate specification by promoting cell proliferation and inhibiting Bmp4 expression. Development 132, 2401-2413.

Merzdorf, C.S., Sive, H.L., 2006. The zic1 gene is an activator of Wnt signaling. Int J Dev Biol 50, 611-617.

Nakade, S., Sakuma, T., Sakane, Y., Hara, Y., Kurabayashi, A., Kashiwagi, K., Kashiwagi, A., Yamamoto, T., Obara, M., 2015. Homeolog-specific targeted mutagenesis in Xenopus laevis using TALENs. In Vitro Cell Dev Biol Anim 51, 879-884.

Takahashi, N., Tochimoto, N., Ohmori, S.Y., Mamada, H., Itoh, M., Inamori, M., Shinga, J., Osada, S., Taira, M., 2005. Systematic screening for genes specifically expressed in the anterior neuroectoderm during early Xenopus development. Int J Dev Biol 49, 939-951.
