# Supplementary material for: Regulation of gene expression downstream of a novel Fgf/Erk pathway during Xenopus development
Source: PLoS One. 2023 Oct 19;18(10):e0286040. doi: 10.1371/journal.pone.0286040 (PMC10586617; doi:10.1371/journal.pone.0286040)
Supplement: Attachment — Submitted filename: Review Manuscript PONE-D-22-31549.pdf [file pone.0286040.s019.pdf]

Review Manuscript PONE-D-22-31549

Cowell and co-workers establish Cic (Capicua) as downstream target of Fgf/Erk signaling in the Xenopus embryo. Cic protein levels are reduced by Fgf signals and wounding-induced Erk activity. Using a TALEN targeting approach the authors show that knockdown of Cic causes gastrulation and primary axis defects. RNA-Seq analysis of embryos injected with FGF4 DNA and Cic-TALEN lead to the identification of common target genes, including the transcription factor fos. Bioinformatic analysis reveals a putative Cic binding site in a conserved intron 1 region of the fos gene. Exposure of early embryos to pharmacological drugs show that fos expression in the mesoderm depends on Fgf/Erk signaling and that wound-induced fos upregulation requires, at least in part, Erk activation. In addition, the transcriptomic analysis identifies ragl11b and atf3 as alternative targets of the Erk-Cic axis at the neural plate border and in response to ectodermal wounding, respectively. Together, Cic acts as a transcriptional repressor of several Fgf/Erk target genes in gastrulation, neural tube closure and wound healing.

The experiments are carefully carried out, the data are convincingly presented, and the manuscript is well written. Publication is recommended in PLOS-One, provided that the following comments are satisfactorily addressed:

1. The authors show nuclear localization of myc-Cic. Nuclear localization signals could be checked by bioinformatic analysis and, if detected, indicated in the Cic protein sequences.

2. The authors show that Cska-FGF4 and wounding decrease the protein levels of myc-Cic in mRNA-injected embryos. It would be interesting to see whether downregulation of FGF/Erk signaling stabilizes myc-Cic protein. A Westernblot analysis of myc-Cic-injected embryos after treatment with SU5402, PD0325901 or DMSO as control could be performed.

3. FGF/Erk signals induce mesoderm in Xenopus embryos. Does Cic knockdown promote mesodermal fate? Cic-TALEN and control embryos at stages 10-14 could be analyzed by whole-mount in situ-hybridization or RT-PCR analysis e.g. with the Xbra marker.

4. The authors show that knockdown of Cic causes blastopore closure, axial and anterior defects. How does overexpression of Cic affect Xenopus development? Can injection of myc-Cic rescue any of the Cic-TALEN phenotypes?

Minor comment:
Christen and Slack (1999. Development) already described dpErk activity at the neural plate border, for which credit should be given.
