# Supplementary material for: Regulation of gene expression downstream of a novel Fgf/Erk pathway during Xenopus development
Source: PLoS One. 2023 Oct 19;18(10):e0286040. doi: 10.1371/journal.pone.0286040 (PMC10586617; doi:10.1371/journal.pone.0286040)
Supplement: Attachment — Submitted filename: Response to Reviewers.docx [file pone.0286040.s020.docx]

Dear Professor Eivers,

Many thanks for the opportunity to resubmit our manuscript [PONE-D-22-31549]. We found the referee comments very useful and address their specific comments in the annotated reviews below. This has entailed quite a lot of reorganisation and textual changes, which, as requested, are highlighted in the attached document. We feel that the changes have significantly improved the manuscript and we hope that you now find it suitable for publication in PLOS ONE.

As requested, we confirm that “The authors have declared that no competing interests exist.”

We look forward to hearing from you regarding this.

Relevant gel photos are now included in supporting data.

With very best regards,

Harry Isaacs (13-4-23)

Journal Requirements:

When submitting your revision, we need you to address these additional requirements.

1. Please ensure that your manuscript meets PLOS ONE's style requirements, including those for file naming. The PLOS ONE style templates can be found at 
<https://journals.plos.org/plosone/s/file?id=wjVg/PLOSOne_formatting_sample_main_body.pdf> and <https://journals.plos.org/plosone/s/file?id=ba62/PLOSOne_formatting_sample_title_authors_affiliations.pdf>

2. Thank you for stating the following in your Competing Interests section:  
"No"

Please complete your Competing Interests on the online submission form to state any Competing Interests. If you have no competing interests, please state ""The authors have declared that no competing interests exist."", as detailed online in our guide for authors at <http://journals.plos.org/plosone/s/submit-now> 
 This information should be included in your cover letter; we will change the online submission form on your behalf.

3. PLOS ONE now requires that authors provide the original uncropped and unadjusted images underlying all blot or gel results reported in a submission’s figures or Supporting Information files. This policy and the journal’s other requirements for blot/gel reporting and figure preparation are described in detail at <https://journals.plos.org/plosone/s/figures#loc-blot-and-gel-reporting-requirements> and <https://journals.plos.org/plosone/s/figures#loc-preparing-figures-from-image-files>. When you submit your revised manuscript, please ensure that your figures adhere fully to these guidelines and provide the original underlying images for all blot or gel data reported in your submission. See the following link for instructions on providing the original image data: <https://journals.plos.org/plosone/s/figures#loc-original-images-for-blots-and-gels>. 
  
In your cover letter, please note whether your blot/gel image data are in Supporting Information or posted at a public data repository, provide the repository URL if relevant, and provide specific details as to which raw blot/gel images, if any, are not available. Email us at [plosone@plos.org](mailto:plosone@plos.org) if you have any questions.

*Response:	Raw uncropped data for Figure 1D is now supplied in Figures_S18 and 18. There is a new western blot in Figure_S3A. Please note that this figure is uncropped and shows the whole of the blot.*

4. We note that you have stated that you will provide repository information for your data at acceptance. Should your manuscript be accepted for publication, we will hold it until you provide the relevant accession numbers or DOIs necessary to access your data. If you wish to make changes to your Data Availability statement, please describe these changes in your cover letter and we will update your Data Availability statement to reflect the information you provide.

*Response:	Genbank accession numbers are now in the text. The RNA-seq data has been submitted to GEO and the series record accession number is now included.*

5. We note that you have included the phrase “data not shown” in your manuscript. Unfortunately, this does not meet our data sharing requirements. PLOS does not permit references to inaccessible data. We require that authors provide all relevant data within the paper, Supporting Information files, or in an acceptable, public repository. Please add a citation to support this phrase or upload the data that corresponds with these findings to a stable repository (such as Figshare or Dryad) and provide and URLs, DOIs, or accession numbers that may be used to access these data. Or, if the data are not a core part of the research being presented in your study, we ask that you remove the phrase that refers to these data.

*Response:	This phrase is no longer used in the text.*

6. Please include captions for your Supporting Information files at the end of your manuscript, and update any in-text citations to match accordingly. Please see our Supporting Information guidelines for more information: <http://journals.plos.org/plosone/s/supporting-information>.


*Response:	Caption text is included.*

Reviewers' comments:

Reviewer's Responses to Questions

**Comments to the Author**

1. Is the manuscript technically sound, and do the data support the conclusions?

The manuscript must describe a technically sound piece of scientific research with data that supports the conclusions. Experiments must have been conducted rigorously, with appropriate controls, replication, and sample sizes. The conclusions must be drawn appropriately based on the data presented.

Reviewer #1: Partly

Reviewer #2: Yes

2. Has the statistical analysis been performed appropriately and rigorously?

Reviewer #1: Yes

Reviewer #2: I Don't Know

3. Have the authors made all data underlying the findings in their manuscript fully available?

The [PLOS Data policy](http://www.plosone.org/static/policies.action#sharing) requires authors to make all data underlying the findings described in their manuscript fully available without restriction, with rare exception (please refer to the Data Availability Statement in the manuscript PDF file). The data should be provided as part of the manuscript or its supporting information, or deposited to a public repository. For example, in addition to summary statistics, the data points behind means, medians and variance measures should be available. If there are restrictions on publicly sharing data—e.g. participant privacy or use of data from a third party—those must be specified.

Reviewer #1: Yes

Reviewer #2: Yes

4. Is the manuscript presented in an intelligible fashion and written in standard English?

PLOS ONE does not copyedit accepted manuscripts, so the language in submitted articles must be clear, correct, and unambiguous. Any typographical or grammatical errors should be corrected at revision, so please note any specific errors here.

Reviewer #1: Yes

Reviewer #2: Yes

5. Review Comments to the Author

Please use the space provided to explain your answers to the questions above. You may also include additional comments for the author, including concerns about dual publication, research ethics, or publication ethics. (Please upload your review as an attachment if it exceeds 20,000 characters)

Reviewer #1: In this paper, the authors examined Xenopus fos gene as a Cic-regulated gene. Based on authors’ hypothesis that Cis downregulation and FGF overexpression showed similar effects on target gene expression, they performed RNAseq analysis and identified genes which is upregulated by both conditions. The authors next analyzed fos gene, one of them, and showed that the expression is seen in Xenopus gastrula in a pattern similar to dpErk activated domain. Furthermore, they found that fos was upregulated by wounding after massive activation of Erk.
The data provided by the authors are fundamentally interesting, but the study purpose is not clear because they first focused CIC but the in the later part they studied about fos, not CIC. In addition, CIC function for FGF signaling have already shown in Drosophila and mammals thus the role for the signaling in Xenopus is easily anticipated from these studies. If the authors want to clarify the function of CIC in Xenopus, the number of experiments is absolutely small (they showed only superficial phenotype in KD embryo). For accepting the paper, I recommend that the authors revise the question (and title) to match the data.

Major comments:
1. Although the title of the paper says “Cic is a mediator ….. in Xenopus development “, the authors showed only KD data, and the later part they carried out a series of experiments regarding fos. The authors should change the title to more appropriate one.

*Response:	As requested we have re-formulated the title to :”* *Regulation of gene expression downstream of a novel Fgf/Erk pathway during Xenopus development”. This removes Cic from the title. We did not include fos in the title as it is used to validate conclusions about a much larger cohort of genes regulated in a similar manner.*

2. P3L58: If they authors aim to reveal that CIC constitutively inhibits target genes in the absence of FGF signaling, they should carry out other experiments (the analysis of target gene expression in CIC OE or KD for example)

3. Fig. 3: as commented above, the authors should add a series of experiment related to CIC function in Xenopus embryo. It is quite difficult to indicate the role of CIC from this data alone.

*Response to point 2 and 3: As requested we have now included a more detailed analysis of gene expression in Cic knockdown embryos using the RNA-seq data (Figure 2D), which is also supported by QPCR data (Figure_S10). We have also included PANTHER gene ontology analyses of targets of the Fgf/Erk/Cic pathway to reveal the types of processes regulated by this pathway (Tables_S6 and 7). These experiments provide a more detailed analysis of Cic function in Xenopus development, lines 253-269. We also include an analysis of Cic overexpression (see referee #2, response 4), lines 215-218.*

4. P8L219/Fig.3B: blastopore defect is also observed in FGF inhibited embryo (as described in author’s previous study). The author should discuss this point.

*Response: Yes, this is a good point and we now address this issue in the text, lines 247-252.*

5. P8L242: Fos gene is well-known target of FGF signaling. If fos is chosen, RNAseq analysis using CIC makes little sense. The author should state the reason more clearly why they focused on fos gene (of course I understand that fos is the most differential gene by CIC-KD/FGF-OE).

*Response: Part of the interest of our observation is that Fos has not been shown to be transcriptionally regulated by Fgf signalling in Xenopus. We address the rationale for our interest more clearly in the text, lines 302-310. We have also included a new panel A in Figure 3 which shows clearly the congruent behaviour of a large cohort of genes in Cic knockdown and Fgf4 overexpressing embryos that provides additional support for our underpinning hypothesis that Fgf4 overexpression and Cic knockdown will have overlapping effects on development.*

6. Readers will want to know other candidates. The author showed the result of rasl11b and atf3 in supplementary figure, but the data should move to main figure.
6. Fig. 4:

*Response: Agreed, this is a good idea we have now moved the rasl11b normal expression into Figure 4 and have added new data regarding rasl11b expression by Fgf/Erk signalling (Figures 4P to V). We have also included the rasl11b and atf3 wounding data in Figures 5I and J.*

7. The authors should confirm that CIC KD actually upregulates fos gene expression by in situ hybridization or RT-PCR.

*Response: QPCR data analysing fos expression are now included (Figure_S10), lines 284_289.*

8. P8L250/Fig. 4B and D: fos expression domain is larger than dpERK positive region. This result does not fully support the claim that fos is activated in the ERK pathway. How can this be interpreted?

*Response: The referee raises an interesting issue and this in now discussed in the text, lines 318-321.*

9. P9L265-6, L267 and L269: the authors refer to the edge of the neural plate as “neural plate border”, but generally “neural plate border“ indicates the anterior region including neural crest and cranial placode. The description should be corrected.

*Response: Good point, this could be confusing we address this in the text and now refer to “the edge of the neural plate”.*

10. Fig. S4K-M: The result that SU treatment did not abolish dpERK activation but PD completely inhibited dpERK activation is a little strange. Actually, the results shown in Fig.4N and O is opposite (the effect of SU treatment on dpErk activation is severer than PD). The authors should at least explain this point.

*Response: We address this in the text Line 346-348. The basic take home is that the Fgfr drug inhibitor has little effect on dpERK activity at the edge of the neural plate whereas the Mek inhibitor blocks it completely. This suggests that fos at the edge of the neural plate is likely to be regulated by Erk but this is independent of Fgf signalling, which is quite an interesting observation.*

Minor comments:
11. P2L29: neural plate plate margin

*Response: As described above this is now addressed*

12. P20L677, L691 Fgf/CiC/Erk ->Fgf/Erk/Cic: Cic/Erk->Erk/Cic

*Response: Thanks for spotting this. We have now corrected.*

13. SupFig1 legend is incorrect (D: Xt CIC-l and human CIC-l). Please check it.

*Response: Now addressed*

14. Fig. S4N are missing.

*Response: Whoops, now corrected in the new figure.*

Reviewer #2: Review Manuscript PONE-D-22-31549

Cowell and co-workers establish Cic (Capicua) as downstream target of Fgf/Erk signaling in the Xenopus embryo. Cic protein levels are reduced by Fgf signals and wounding-induced Erk activity. Using a TALEN targeting approach the authors show that knockdown of Cic causes gastrulation and primary axis defects. RNA-Seq analysis of embryos injected with FGF4 DNA and Cic-TALEN lead to the identification of common target genes, including the transcription factor fos. Bioinformatic analysis reveals a putative Cic binding site in a conserved intron 1 region of the fos gene. Exposure of early embryos to pharmacological drugs show that fos expression in the mesoderm depends on Fgf/Erk signaling and that wound-induced fos upregulation requires, at least in part, Erk activation. In addition, the transcriptomic analysis identifies ragl11b and atf3 as alternative targets of the Erk-Cic axis at the neural plate border and in response to ectodermal wounding, respectively. Together, Cic acts as a transcriptional repressor of several Fgf/Erk target genes in gastrulation, neural tube closure and wound healing.

The experiments are carefully carried out, the data are convincingly presented, and the manuscript is well written. Publication is recommended in PLOS-One, provided that the following comments are satisfactorily addressed:

1. The authors show nuclear localization of myc-Cic. Nuclear localization signals could be checked by bioinformatic analysis and, if detected, indicated in the Cic protein sequences.

*Response: Now discussed in text and shown on Figure_S1, line 191 and lines 447-449.*

2. The authors show that Cska-FGF4 and wounding decrease the protein levels of myc-Cic in mRNA-injected embryos. It would be interesting to see whether downregulation of FGF/Erk signaling stabilizes myc-Cic protein. A Western blot analysis of myc-Cic-injected embryos after treatment with SU5402, PD0325901 or DMSO as control could be performed.

*Response: We agree that this might be interesting, but the referee does not indicate that this is essential before publication and feel that another experiment with overexpressed tagged protein would not add much to the current story. It would be even more interesting to carry out the suggested experiment and look for changes in the endogenous Cic protein. However, in the absence of a Xenopus Cic antibody we are unable to do this and as such, is beyond the scope of the current study.*

3. FGF/Erk signals induce mesoderm in Xenopus embryos. Does Cic knockdown promote mesodermal fate? Cic-TALEN and control embryos at stages 10-14 could be analyzed by whole-mount in situ-hybridization or RT-PCR analysis e.g. with the Xbra marker.

*Response: As requested we have now included a more detailed analysis of marker gene expression in Cic knockdown embryos using the RNA-seq data (Figure 2D), which is also supported by QPCR data (Figure_S10). This shows that there is little effect on the expression Xbra(Tbxt) suggesting that Cic knockdown does not promote mesodermal fate per se.*

4. The authors show that knockdown of Cic causes blastopore closure, axial and anterior defects. How does overexpression of Cic affect Xenopus development? Can injection of myc-Cic rescue any of the Cic-TALEN phenotypes?

*Response: We now include a figure (Figure S4) showing that Cic overexpression has no obvious effects on development, lines 215-219.*

Minor comment:
Christen and Slack (1999. Development) already described dpErk activity at the neural plate border, for which credit should be given.

*Response: Yes, an oversight on our part this is now addressed in the text.*

6. PLOS authors have the option to publish the peer review history of their article ([what does this mean?](https://journals.plos.org/plosone/s/editorial-and-peer-review-process#loc-peer-review-history)). If published, this will include your full peer review and any attached files.

If you choose “no”, your identity will remain anonymous but your review may still be made public.


**Do you want your identity to be public for this peer review?** For information about this choice, including consent withdrawal, please see our [Privacy Policy](https://www.plos.org/privacy-policy).

Reviewer #1: No

Reviewer #2: No

[NOTE: If reviewer comments were submitted as an attachment file, they will be attached to this email and accessible via the submission site. Please log into your account, locate the manuscript record, and check for the action link "View Attachments". If this link does not appear, there are no attachment files.]

While revising your submission, please upload your figure files to the Preflight Analysis and Conversion Engine (PACE) digital diagnostic tool, <https://pacev2.apexcovantage.com/>. PACE helps ensure that figures meet PLOS requirements. To use PACE, you must first register as a user. Registration is free. Then, login and navigate to the UPLOAD tab, where you will find detailed instructions on how to use the tool. If you encounter any issues or have any questions when using PACE, please email PLOS at [figures@plos.org](mailto:figures@plos.org). Please note that Supporting Information files do not need this step.
