# Supplementary material for: Regulation of gene expression downstream of a novel Fgf/Erk pathway during Xenopus development
Source: PLoS One. 2023 Oct 19;18(10):e0286040. doi: 10.1371/journal.pone.0286040 (PMC10586617; doi:10.1371/journal.pone.0286040)
Supplement: Attachment — Submitted filename: Response to reviewers 2-5-23.docx [file pone.0286040.s021.docx]

Dear Professor Eivers,

As requested, we have addressed the minor issues raised by the reviewers (please see comments below). we hope that you now find it suitable for publication in PLOS ONE.

We look forward to hearing from you regarding this.

With very best regards,

Harry Isaacs (02-05-23)

==================================================================================

Reviewer #1: The authors responded appropriately to the reviewers' comments. The addition of RT-PCR to Fig. 2D provides a more detailed description of what genes are affected by CiC KD. And results for other genes (rasl11, atf3) were added to the main figures, reducing the dissociation between the RNA-seq using CiC KD/Fgf4 OE embryos and the experiments in the later part. Other points have also been described in more detail. The revised manuscript was greatly improved.

Minor:
1)L376: In Fig. 3D/E, which dot shows atf3? It would be better to show it here.

*Response: This is now addressed in the text (See line 377).*

Reviewer #2: The authors have now satisfactorily addressed all my comments! The new experimental data, including the demonstration that Cic knockdown has little effect on Xbra/tbxt expression (Fig. 2D, Fig. S10) and that gfp-Cic overexpression does not affect Xenopus development (Fig. S3) contribute to a more comprehensive understanding of the role of this protein. The identification and indication of a nuclear localization signal in the Cic protein (Fig. S1) is appreciated.

No further experiments are expected but the following points should be addressed before the manuscript can be accepted:


1. Lines 468-9. As far as I can see, no experimental evidence is given that supports the conclusion “Our data indicate that later expression domains of fos in the lateral edges of the neural plate … are not regulated by Fgf signalling.” Please correct!

*Response: Sentence now deleted. Line 469*

2. Fig. 1F shows that wounding induces a decrease in Cic-myc protein levels. Please clarify how long the embryos were incubated after wounding before they were harvested for Western blot analysis!

*Response: Timing now included in figure legend. Line786-787*

3. The new bar chart in in Fig. 2D helps to follow the Cip-Talen phenotype! For the sake of clarity, a subtitle for this panel such as “Cic-knockdown versus control” could be added within the figure. In addition, “Cic-Talen” should be indicated in panels A-C.

*Response: Figure 2 now has subtitles for all panels*

4. Line 352: Correct “the expression of domain of ras11b”!

*Response: Now corrected*

5. Line 965: Figure 16 > Figure S16.

*Response: Now corrected. Line 966*
